# iDDN: determining trans-omics network structure and rewiring with integrative differential dependency networks

**DOI:** 10.1093/bioadv/vbaf086

**Published:** 2025-05-08

**Authors:** Yizhi Wang, Yi Fu, Yingzhou Lu, Zhen Zhang, Robert Clarke, Sarah J Parker, David M Herrington, Guoqiang Yu, Yue Wang

**Affiliations:** The Bradley Department of Electrical and Computer Engineering, Virginia Polytechnic Institute and State University, Arlington, VA 22203, United States; The Bradley Department of Electrical and Computer Engineering, Virginia Polytechnic Institute and State University, Arlington, VA 22203, United States; The Bradley Department of Electrical and Computer Engineering, Virginia Polytechnic Institute and State University, Arlington, VA 22203, United States; Department of Pathology, Johns Hopkins Medical Institutions, Baltimore, MD 21231, United States; The Hormel Institute, University of Minnesota, Austin, MN 55912, United States; Smidt Heart Institute, Cedars-Sinai Medical Center, West Hollywood, CA 91001, United States; Department of Internal Medicine, Wake Forest University, Winston-Salem, NC 27157, United States; Department of Automation, Tsinghua University, Beijing 100084, P.R. China; The Bradley Department of Electrical and Computer Engineering, Virginia Polytechnic Institute and State University, Arlington, VA 22203, United States

## Abstract

**Motivation:**

Mapping the gene networks that drive disease progression allows identifying molecules that rectify the network by normalizing pivotal regulatory elements. Upon mechanistic validation, these upstream normalizers represent attractive targets for developing therapeutic interventions to prevent the initiation or interrupt the pathways of disease progression. Differential network analysis aims to detect significant rewiring of regulatory network structures under different conditions. With few exceptions, most existing tools are limited to inferring differential networks from single-omics data that could be incomplete and prone to collapse when trans-omics multifactorial regulatory mechanisms are involved.

**Results:**

We previously developed an efficient differential network analysis method—Differential Dependency Networks (DDN), that enables joint learning of common network structure and rewiring under different conditions. We now introduce the integrative DDN (iDDN) tool that extends this framework with biologically principled designs to make robust multi-omics differential network inferences. The comparative experimental evaluations on both realistic simulations and case studies show that iDDN can help biologists more accurately identify, in a study-specific and often unknown trans-omics regulatory circuitry, a network of differentially wired molecules potentially responsible for phenotypic transitions.

**Availability and implementation:**

The Python package of iDDN is available at https://github.com/cbil-vt/iDDN. A user’s guide is provided at https://iddn.readthedocs.io/.

## 1 Introduction

Traditional differential expression analysis often overlooks genes with modest expression shifts but crucial roles in cellular responses, due to its focus on large expression changes (Hu *et al.* 2016, Nouri *et al.* 2024). In contrast, mapping the gene regulatory networks (GRNs) empowers identifying molecules that may rectify the pivotal regulatory elements, rather than targeting peripheral downstream effectors (Theodoris *et al.* 2023, Cui *et al.* 2024) These insights have driven the development of differential network analysis, which searches for changes in the regulatory network wiring diagram that are associated with disease progression (Kadelka and Murrugarra 2024). For example, several networks of differentially connected molecules under different conditions were identified based on study-specific and often unknown baseline regulatory circuitries (Zhang *et al.* 2009, Mitra *et al.* 2013, Nouri *et al.* 2024). By focusing on condition-driven structural changes, these topological signature molecules represent logical targets for normalizing pivotal regulatory circuits associated with disease progression, due to their potentially profound downstream effects within gene networks (Zhang *et al.* 2016, Herrington *et al.* 2018, Fu *et al.* 2024).

Mapping the gene network structure requires multi-omics data acquired from the same samples and an integrative network inference framework that can represent true associations between multi-omics regulators and effectors in a graph. Most previous approaches for regulatory network inference have relied on correlation metrics to measure the co-expressions of molecule nodes in a network (Mitra *et al.* 2013, Kamimoto *et al.* 2023). However, these methods are limited to marginal correlation networks that are estimated using univariate analysis and do not distinguish between direct and indirect relationships. As a result, theoretically, this limitation will lead to transitive connections and false-positive edges and generally cannot infer true network structures (Zhang *et al.* 2009, Nouri *et al.* 2024). This drawback has been addressed in the Gaussian Graphical Model (GGM) framework, which explicitly characterizes the conditional dependence among variables of the corresponding network structure (Meinshausen and Bühlmann 2006). The GGM framework has since been further advanced through methods like fused-Lasso Differential Dependency Network (DDN) and Joint Graphical Lasso (JGL), which are designed to detect the rewiring of network structure under different conditions (Zhang and Wang 2010, Danaher *et al.* 2014, Fu *et al.* 2024). Nevertheless, with few exceptions, most existing tools remain limited to inferring differential networks from single-omics data. This limitation renders them incomplete and prone to collapse when addressing the multifactorial regulatory mechanisms inherent in trans-omics studies (Class *et al.* 2018, Yuan and Duren 2025).

**Figure 1.**
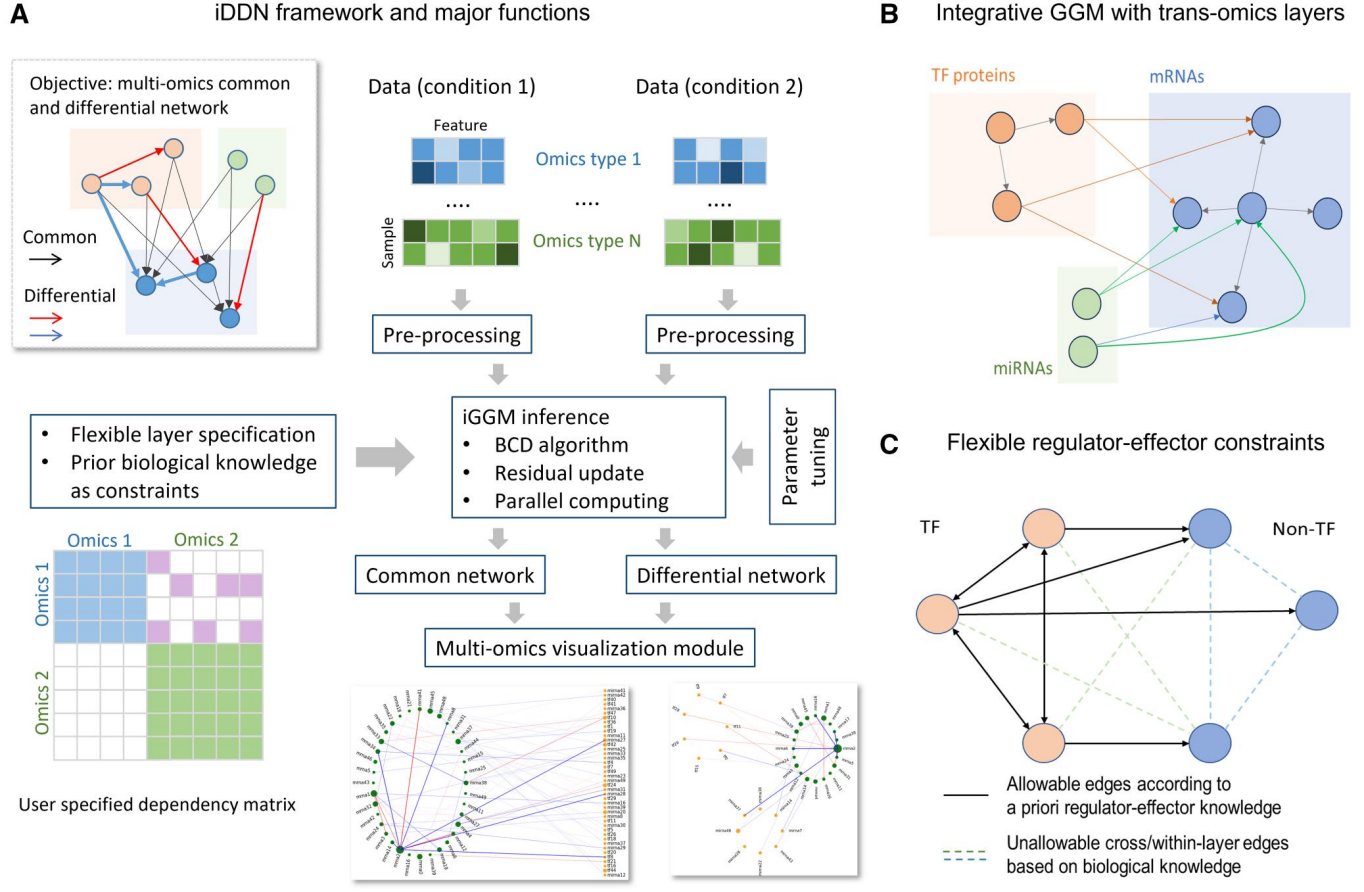
Overview of the iDDN workflow and key features. (A) iDDN workflow. iDDN is designed to accurately and efficiently infer common and differential networks (top left) from multi-omics data under two conditions (top right). It is a user-friendly Python package that enables users to define omics layers, incorporate prior constraints (bottom left), efficiently infer networks, tune hyperparameters, and visualize the results (bottom right). (B) Multi-omics data integration. iDDN models relationships among molecules both within and across multiple omics layers. The figure illustrates a network integrating three types of omics data. (C) Incorporating prior constraints. iDDN allows for the inclusion of prior known regulatory relationships as constraints in the optimization process. The example highlights the specification of known TF-target gene relationships as allowable edges in iDDN.

Here, we introduce integrative DDN (iDDN), an open-source software tool with a biologically principled design to support multi-omics differential network inferences. To accomplish this, we improved our previously developed DDN tools, which utilize fused Lasso regressions to jointly learn common and rewired network structures (Tian *et al.* 2014, Fu *et al.* 2024), by incorporating three novel and practical functional designs: (i) a concatenated GGM with trans-omics layers to represent intra-omics and inter-omics associations, (ii) feasible and flexible application of modeling constraints to comply with a priori regulator-effector relationships, and (iii) integrative algorithmic acceleration strategies to make accurate and robust inference of multi-omics network structure and rewiring (Fig. 1). We demonstrate the effectiveness and utility of these new features in iDDN through both realistic simulations and biomedical case studies, showing improved accuracy in differential network analysis compared to benchmark methods.

## 2 Methods

After a brief review of the DDN framework, we introduce the iDDN framework, its acceleration strategies, and considerations for hyper-parameter tuning. More details can be found in Supplementary Section S1.

### 2.1 Review of the DDN framework

Here, we consider the problem of simultaneously learning the common structure and the rewiring of a GGM between two conditions. Consider the same set of p molecular variables under two conditions, with sample sizes *N*_1_ and *N*_2_, and let the data matrices be denoted as $X^{\left(1\right)}$ and $X^{\left(2\right)}$. We formulate DDN inference using fused Lasso regression (Zhang and Wang 2010, Fu *et al.* 2024). Specifically, for node (molecule) i, under each condition, we treat all other nodes as predictors and denote the coefficients as $\beta_{i}^{\left(1\right)}$ and $\beta_{i}^{\left(2\right)}$, respectively. In addition, we impose sparsity penalties on $\beta_{i}^{\left(1\right)}$ and $\beta_{i}^{\left(2\right)}$, as well as their differences. The objective function for each node i=1, 2, …, p is defined as

(1) $$L_{\mathrm{DDN}}(i)=L_{\mathrm{data}}+L_{\mathrm{sparsity}}+L_{\mathrm{similarity}}.\;$$

Here, $L_{\mathrm{data}}$ is the data term about regression error:

$$L_{\mathrm{data}}=\frac{1}{N_{1}}{\left\|y_{i}^{\left(1\right)}-X^{\left(1\right)}\beta_{i}^{\left(1\right)}\right\|}_{2}^{2}+\frac{1}{N_{2}}{\left\|y_{i}^{\left(2\right)}-X^{\left(2\right)}\beta_{i}^{\left(2\right)}\right\|}_{2}^{2},$$

where $y_{i}^{\left(1\right)}$ and $y_{i}^{\left(2\right)}$ denote the observations of node i under the two conditions. Additionally, the *i*th elements of $\beta_{i}^{\left(1\right)}$ and $\beta_{i}^{\left(2\right)}$ are set to 0, as we do not model self-regulation. The $L_{\mathrm{sparsity}}$ term enforces sparsity of coefficients:

$$L_{\mathrm{sparsity}}=\lambda_{1}\sum_{j=1}^{p}\left(\left|\beta_{i,j}^{\left(1\right)}|+|\beta_{i,j}^{\left(2\right)}\right|\right),$$

while $L_{\mathrm{similarity}}$ encourages the similarity of networks under two conditions:

$$L_{\mathrm{similarity}}=\lambda_{2}{\left\|\beta_{i}^{\left(1\right)}-\beta_{i}^{\left(2\right)}\right\|}_{1}.$$

Here, $\lambda_{1}$ and $\lambda_{2}$ are the *L*_1_ regularization hyperparameters on common and differential edges, respectively. The goal is to solve $\beta_{i}=(\beta_{i}^{\left(1\right)},\beta_{i}^{\left(2\right)})$ that minimizes $L_{\mathrm{DDN}}(i)$ for each node. If the regression coefficient $\beta_{ij}$ from node j to node i is zero, the two variables are conditionally independent given the observations of other nodes. Conversely, a nonzero $\beta_{ji}$ indicates that node j and i are not conditionally independent, and an edge is drawn between them in the resulting network. The $L_{\mathrm{sparsity}}$ term enables the inference of a sparse common graph structure, while the $L_{\mathrm{similarity}}$ term ensures reliable detection of sparse network rewiring between two conditions, reducing false positives caused by data inconsistencies.

### 2.2 iDDN framework

Network-based multi-omics data integration aims to infer associations among molecular features within and across platforms, providing a more complete and mechanistic understanding of gene regulatory ecosystems (Fig. 1B). To capture information across multiple intra-omics layers, we designed the iDDN framework using a layered trans-omics GGM to infer common network structure and dependency rewiring within and between layers under different conditions (Fig. 1A). When *a priori* regulator-effector relationships are known, iDDN can flexibly and readily incorporate them as biologically plausible constraints (Fig. 1C).

In DDN, the goal is to estimate the coefficients for node i under two conditions, denoted as $\beta_{i}^{\left(1\right)}$ and $\beta_{i}^{\left(2\right)}$. In iDDN, the major difference is that for node i, we allow it to depend only on a specific set of nodes A(i), which imposed binary choices between predictors and dependent variables. In DDN, the set of predictors always includes all other nodes, such that A(i)={1,…,i−1,i+1,…p}. In contrast, iDDN allows users to specify A(i) for each node, indicating which nodes are permitted to connect to node i. As a result, A(i)⊂{1,…,i−1,i+1,p}. For clarity, we use $\beta_{i,A(i)}^{\left(1\right)}$ and $\beta_{i,A(i)}^{\left(2\right)}$ to denote the coefficients we aim to estimate for node i in iDDN. We formulate iDDN as a convex optimization problem, with the following objective function:

(2) $$L_{\mathrm{iDDN}}(i)=L_{\mathrm{data}}+L_{\mathrm{sparsity}}+L_{\mathrm{similarity}}.\;$$

Here, the data term is defined as follows:

$$L_{\mathrm{data}}=\frac{1}{N_{1}}{\left\|y_{i}^{\left(1\right)}-X_{A(i)}^{\left(1\right)}\beta_{i,A(i)}^{\left(1\right)}\right\|}_{2}^{2}+\frac{1}{N_{2}}{\left\|y_{i}^{\left(2\right)}-X_{A(i)}^{\left(2\right)}\beta_{i,A(i)}^{\left(2\right)}\right\|}_{2}^{2}.$$

Here, $X_{A(i)}^{\left(1\right)}$ is the columns corresponding to A(i) in the observed data under the first condition, and $X_{A(i)}^{\left(2\right)}$ is similarly defined. Next, we have

$$L_{\mathrm{sparsity}}=\sum_{j\in A(i)}^{}\lambda_{1}(i,j)(\left|\beta_{i,j}^{\left(1\right)}|+|\beta_{i,j}^{\left(2\right)}\right|).$$

Here, $\beta_{i,j}^{\left(1\right)}$ is the coefficient for the molecule j. Unlike DDN, iDDN allows each pair of molecules to have different sparsity constraints, which is useful when modeling layers with distinct biological properties. The $L_{\mathrm{similarity}}$ term is

$$L_{\mathrm{similarity}}=\sum_{j\in A(i)}^{}\lambda_{2}(i,j)|\beta_{i,j}^{\left(1\right)}-\beta_{i,j}^{\left(2\right)}|.$$

This term permits different similarity penalties for each edge, which is useful when some layers exhibit higher consistency between conditions, while others are expected to have more frequent rewiring events.

We note that iDDN standardizes the data such that each feature under each condition has a mean of zero and a unit variance. As a result, users need to filter out features with zero variance in either condition. Typically, this step is part of broader feature selection processes, which depend on specific data characteristics and biological questions.

### 2.3 Specifying constraints in iDDN

The introduction of the dependent set A(i) in the iDDN framework simplifies the integration of layers of omics data and allows flexible specification of the relationships between layers. For instance, biological constraints can be readily applied to align with the central dogma or other known regulatory mechanisms by appropriately specifying which molecules are permitted to connect to each molecule. Additionally, iDDN facilitates the incorporation of constraints across molecules both within and between layers. If the specified constraints between node i and j are directional (e.g. i→j), we assign i∈A(j) and j∉A(i). This accommodates known regulatory directions between molecules.

To illustrate these ideas, we use a two-layer toy example with three transcription factors (TFs) and three (non-TF) target genes (Fig. 1C). Based on regulatory knowledge, we assume that there are edges among TFs and edges from TFs to targets, but no edges exist among the non-TF genes. We can easily encode this knowledge into A(i). Assuming the first three nodes represent TFs, and the remaining three represent targets, we specify: A(1)={2, 3}, A(2)={1, 3}, and A(3)={1,2}. Here i∉A(i) as self-regulatory effects are not modeled, and nodes beyond 3 are excluded since TFs are not regulated by non-TF genes. For nodes 4 to 6 (the targets), we set A(i)={1, 2, 3}, i=4,…,6, indicating each gene can only be regulated by TFs.

### 2.4 BCD algorithm for iDDN

We adapted and modified the Block Coordinate Descent (BCD) algorithm to minimize the iDDN objective function $L_{\mathrm{iDDN}}$ by jointly learning $\beta_{i,A(i)}^{\left(1\right)}$ and $\beta_{i,A(i)}^{\left(2\right)}$. In the BCD algorithm, at each step, we update a pair of variables corresponding to a molecule under two conditions: $\beta_{i,j}^{\left(1\right)}$ and $\beta_{i,j}^{\left(2\right)}$, j∈A(i). During this step, all other nodes are fixed. In the subsequent step, another molecule (e.g. j+1) is updated, and the process cycles through all nodes until convergence. Convergence is evaluated based on the mean absolute change of the variables, with a threshold of $10^{-6}$. Once convergence is achieved for node i, the algorithm proceeds to the next node (i+1), until all nodes 1,…,p are processed. During each step of the BCD algorithm, the objective function for node i when updating node j, is defined as:

(3) $$L_{\mathrm{iDDN}}{\left(i\right)}_{j}=L_{\mathrm{data},j}+L_{\mathrm{sparsity},j}+L_{\mathrm{similarity},j},$$

where the subscript j emphasizes that node j is being updated in this step. The data term is defined as:

$$L_{\mathrm{data},j}=\sum_{c=1}^{2}\frac{1}{N_{c}}{\left\|y_{i}^{\left(c\right)}-X_{A(i)\backslash j}^{\left(c\right)}\beta_{i,A(i)\backslash j}^{\left(c\right)}-X_{j}^{\left(c\right)}\beta_{i,j}^{\left(c\right)}\right\|}_{2}^{2},$$

where c denotes each condition, A(i)\j refers to all nodes in A(i) except j, and $X_{j}^{\left(c\right)}$ represents observed data for node j under condition c. We define

$$L_{\mathrm{sparsity},j}=\lambda_{1}(i,j)(\left|\beta_{i,j}^{\left(1\right)}|+|\beta_{i,j}^{\left(2\right)}\right|)+\sum_{k\in A(i)\backslash j}^{}\lambda_{1}(i,k)(\left|\beta_{i,k}^{\left(1\right)}|+|\beta_{i,k}^{\left(2\right)}\right|).$$

As for the similarity term, we have

$$L_{\mathrm{similarity},j}=\lambda_{2}(i,j)|\beta_{i,j}^{\left(1\right)}-\beta_{i,j}^{\left(2\right)}|+\sum_{k\in A(i)\backslash j}^{}\lambda_{2}(i,k)|\beta_{i,k}^{\left(1\right)}-\beta_{i,k}^{\left(2\right)}|$$

If we define the residual as $y_{i,\mathrm{resi}}^{\left(c\right)}=y_{i}^{\left(c\right)}-X_{A(i)\backslash j}^{\left(c\right)}\beta_{i,A(i)\backslash j}^{\left(c\right)}$, and ignore terms in $L_{\mathrm{sparsity},j}$ and $L_{\mathrm{similarity},j}$ that are irrelevant to $\beta_{i,j}^{\left(1\right)}$ and $\beta_{i,j}^{\left(2\right)}$, the simplified objective function becomes:

(4) $${\tilde{L}}_{\mathrm{iDDN}}{\left(i\right)}_{j}=\sum_{c=1}^{2}\left[\frac{1}{N_{c}}{\left\|y_{i,\mathrm{resi}}^{\left(c\right)}-X_{j}^{\left(c\right)}\beta_{i,j}^{\left(c\right)}\right\|}_{2}^{2}+\lambda_{1}(i,j)|\beta_{i,j}^{\left(c\right)}|\right]+\lambda_{2}(i,j)|\beta_{i,j}^{\left(1\right)}-\beta_{i,j}^{\left(2\right)}|.\;$$

Since feature in $X^{\left(c\right)}$ are standardized, the optimal values of $\beta_{i,j}^{\left(1\right)}$ and $\beta_{i,j}^{\left(2\right)}$ can be derived in closed form depending on the relationship between $y_{i,\mathrm{resi}}^{c}$, $X_{j}^{c}$, $\lambda_{1}(i,j)$, and $\lambda_{2}(i,j)$, which can be categorized into 13 distinct conditions (Zhang and Wang 2010). The iDDN framework also incorporates additional strategies to accelerate computation. More details can be found in Supplementary Section S1.

### 2.5 Hyper-parameter tuning

The results of iDDN depend on the choice of hyper-parameters $\lambda_{1}$ and $\lambda_{2}$. While the iDDN package provides several methods for hyper-parameter tuning, we encourage users to utilize their domain knowledge to verify and interpret the results, rather than overly relying on automated parameter selection methods. In this section, we discuss cross-validation grid search functions; additional methods are detailed in Supplementary Section S1. By default, we perform repeated 5-fold cross validation 10 times (Pedregosa *et al.* 2011). The iDDN package supports both a 2D grid search for combinations of $\lambda_{1}$ and $\lambda_{2}$, and a sequential search (first determining $\lambda_{1}$, followed by $\lambda_{2}$). For a given pair of $\lambda_{1}$ and $\lambda_{2}$, we apply iDDN to a randomly selected training subset of the dataset (80% by default) to infer the networks. For each node under each condition, the relative reconstruction error is computed on the test subset (20% by default) using the inferred network. Specifically, after binarizing the iDDN inferred $\beta_{i,A(i)}^{\left(c\right)}$, we perform linear regression to re-estimate the regression coefficients using the selected features without a sparsity penalty. This re-estimation step is necessary to avoid the impact of $\lambda_{1}$ on the coefficients. Using these regression coefficients, we estimate the values of the test samples, denoted as $x_{\mathrm{est}}$. The relative error for node i under condition c is calculated as

$${\mathrm{err}}_{i}^{\left(c\right)}=\frac{\mathrm{var}(x_{\mathrm{est}}-x_{\mathrm{truth}})}{\mathrm{var}(x_{\mathrm{truth}})},$$

where $x_{\mathrm{truth}}$ represents the ground truth values for the test samples. The final error for a pair of $\lambda_{1}$ and $\lambda_{2}$ is obtained by averaging the errors across all nodes and conditions. The optimal values of $\lambda_{1}$ and $\lambda_{2}$ can then be selected either by directly using the pair corresponding to the minimum error, or by applying the 1-standard error rule (Friedman *et al.* 2017).

## 3 Validation of iDDN accuracy and efficiency

We first introduce synthetic datasets and evaluation criteria, followed by a demonstration of the benefits of incorporating multiple omics types and prior constraints. Next, we present a comparative evaluation with peer methods and analyze the computational cost of iDDN using a real single-cell multi-omics dataset. An ablation study of iDDN is provided in Supplementary Section S5.

### 3.1 Experiment setup and evaluation criteria

To simulate GRNs, we created a scale-free, three-layer GGM consisting of 50 mRNAs, 50 TF proteins, and 50 miRNAs (Zhang *et al.* 2016). Using this network as a template, we generated networks under two conditions by randomly removing 25% of edges in each instance, with 200 samples generated for each condition. The accuracy of network inference was evaluated by four metrics: partial Receiver Operating Characteristic (pROC) curves for the common dependency network (CDN), $F_{1}$ scores for the CDN and the differential dependency network (DDN), and the average $F_{1}$ score. The $F_{1}$ score is defined as the harmonic mean of precision and recall. Given that iDDN relies on the selection of hyperparameters $\lambda_{1}$ and $\lambda_{2}$, we evaluated iDDN and peer methods using 640 combinations of these parameters to minimize the influence of hyperparameter selection. Each combination was repeated 30 times. For each value of $\lambda_{1}$, we selected the $\lambda_{2}$ value corresponding to the highest $F_{1}$ score. The pROC and $F_{1}$ curves for varying $\lambda_{1}$ values were then plotted. Additional details are provided in Supplementary Section S2.

### 3.2 Impacts of layer numbers and prior constraints

**Figure 2.**
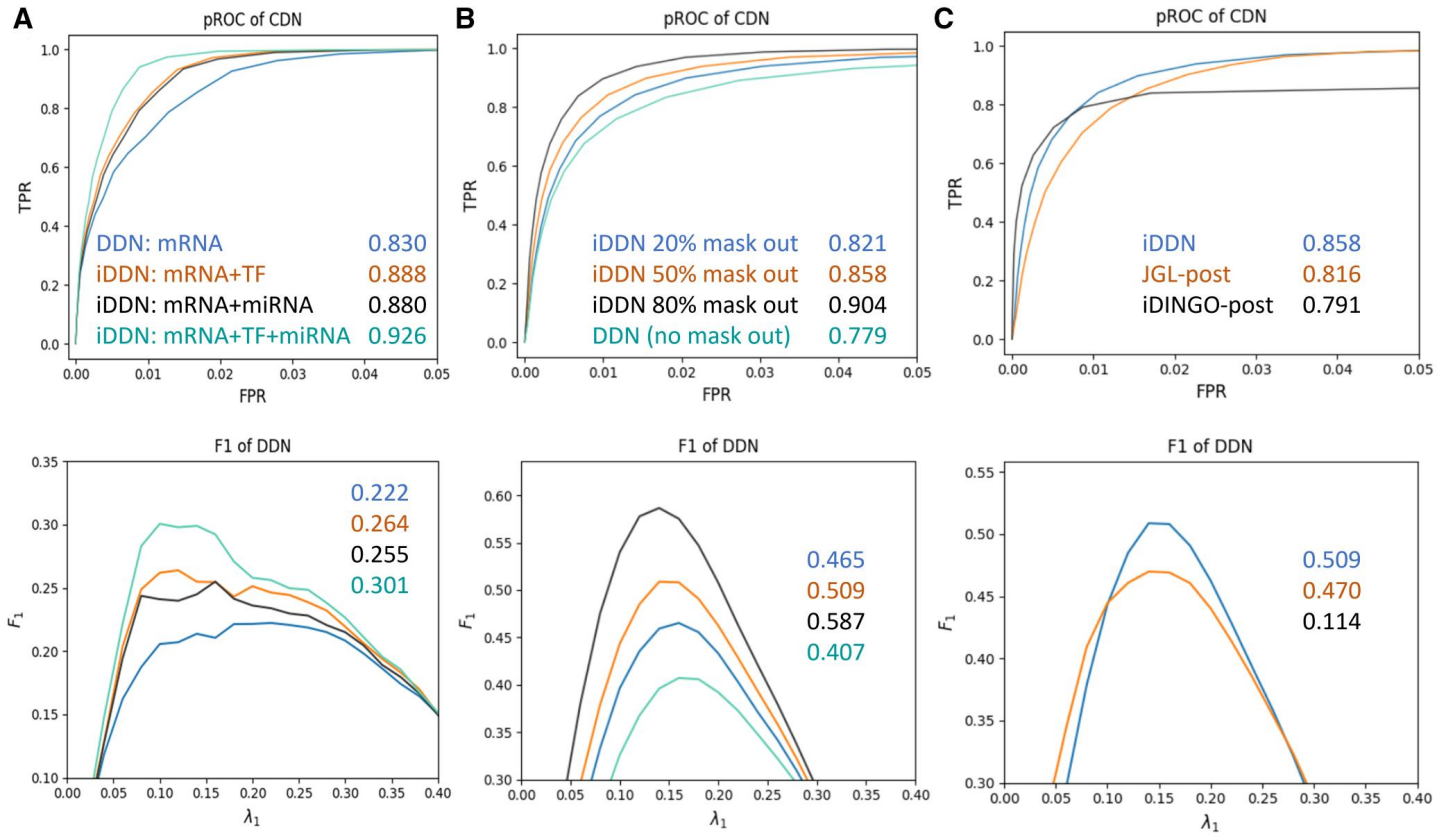
Simulation studies of iDDN. (A) Integration of multiple layers improves the estimation accuracy of mRNA layer. Each line represents one combination of layers. For the first case, results using DDN3.0 are shown. Top: Partial Receiver Operating Characteristic (pROC) curves for estimating the common network between two conditions. These curves are generated by varying the $\lambda_{1}$ values, with $\lambda_{2}$ selected to achieve the best $F_{1}$ score for each $\lambda_{1}$. The annotated number represents the area under the pROC curve [normalized by the false positive rate (FPR) cutoff]. Bottom: $F_{1}$ scores versus $\lambda_{1}$ values for estimating the differential network. The highest $F_{1}$ score for each method is annotated, corresponding to the order in the top figure. (B) Incorporation of prior constraints enhances performance. A higher “mask out” ratio corresponds to stronger constraints. Results from DDN3.0 are shown when no constraints are applied. Top: pROC results for common network inference. Bottom: Results for differential network inference. (C) Comparison of iDDN, JGL, and iDINGO. Constraints used by iDDN are post-applied to JGL and iDINGO. Top: pROC results for common network inference, Bottom: Differential network results. Note that the differential network results for iDINGO fall below the *y*-axis range in the bottom figures.

We validated the performance of iDDN by progressively incorporating additional data types and evaluating the inference accuracy of the mRNA network (Fig. 2A). Specifically, we considered four combinations of layers: (i) mRNA layer only, (ii) mRNA + TF protein, (iii) mRNA + miRNA, and (iv) mRNA + TF protein + miRNA. We simply applied DDN3.0 for the first case. The evaluation was performed on the mRNA layer only. For this analysis, we examined a scenario where each TF protein or miRNA was connected to five mRNAs. The top panel of Fig. 2A illustrates the pROC of common network estimation, demonstrating that incorporating more data types resulted in improved performance. The bottom panel presents the $F_{1}$ scores across different $\lambda_{1}$ values when estimating the differential network, where a higher $F_{1}$ score indicates better performance. Consistently, the inclusion of additional omics types led to enhanced accuracy. When compared to the results for the common network, incorporating multiple omics types—particularly when using both the TF protein and miRNA layers—provided even greater benefits for differential network estimation. This improvement is likely due to the increased challenges associated with estimating differential networks, where additional information yields more pronounced advantages. Overall, these results confirm that iDDN effectively integrates multi-omics data, significantly enhancing the inference accuracy of both common and differential network structures.

We also evaluated the impact of imposing *a priori* regulator-effector constraints by varying the percentage of search space reduction. Specifically, we masked different proportions (0%, 20%, 50%, and 80%) of edges that were not true edges, with stronger constraints corresponding to a higher percentage of masked edges. When 0% of edges were masked, we simply applied DDN3.0. Unlike the previous experiment, this evaluation was performed using all layers and edges. The results, in which each regulator was connected to five targets, are presented in Fig. 2B. Accuracy in estimating both the common and differential networks improved with stronger constraints. The benefits of imposing constraints were especially pronounced for differential networks. These findings demonstrate that iDDN effectively leverages prior knowledge to enhance inference accuracy. Additional results can be found in Supplementary Section S3.

### 3.3 Comparative evaluation with peer methods

We compared the accuracy of iDDN with two most relevant peer methods: JGL (Danaher *et al.* 2014) and iDINGO (Class *et al.* 2018). Similar to iDDN, JGL learns two GGMs under two conditions and requires two hyper-parameters, $\lambda_{1}$ and $\lambda_{2}$. Therefore, for a fair evaluation, we used 640 combinations of hyperparameters. The precision matrices obtained from JGL were converted into common and differential networks for performance evaluation. When using iDINGO, to better adapt to its framework, we combined two regulatory layers into one layer and treated the mRNA layer as the second layer. By default, iDINGO scans a range of $\lambda_{1}$ values to estimate the common network. However, since it did not always select the optimal $\lambda_{1}$, we adjusted the comparison to be fairer by using $\lambda_{1}$ values that worked well with iDDN. Then we set a range of thresholds on iDINGO scores matrices to generate corresponding common and differential networks. For this simulation, we imposed constraints that masked 50% of edges that were not true. To ensure fair comparisons, we enhanced the performance of JGL and iDINGO by post applying the constraints on their results. Specifically, all edges disallowed by the constraints were removed. We refer to these post-processed versions as JGL-post and iDINGO-post.

We compared iDDN, JGL-post, and iDINGO-post in a scenario where each regulator was connected to five mRNAs (Fig. 2C). For iDDN and JGL-post, as in previous analyses, each data point corresponds to a $\lambda_{1}$ value, with the corresponding $\lambda_{2}$ chosen to achieve the highest $F_{1}$ score. For iDINGO-post, each point represents a threshold applied to the estimated common or differential score matrix. In this simulation, iDDN outperformed both JGL-post and iDINGO-post in estimating both common and differential networks. Although iDINGO performed well for common networks—attributable to its use of graphical lasso on the combined data—it did not perform as well for detecting differential edges. Overall, the results demonstrated that iDDN provided more accurate network inference than the peer methods. Additional results are presented in Supplementary Section S3.

### 3.4 Evaluation of running time and memory usage

We evaluated the computational cost of iDDN and peer methods using a publicly available single-cell ATAC+RNA sequencing dataset (10x Genomcis 2010). The evaluations were conducted on a workstation equipped with an Intel Xeon E5-2630 v3 CPU, 128GB RAM, and running Ubuntu 24.04. Unless otherwise stated, all comparisons used $\lambda_{1}\;$= 0.05 and $\lambda_{2}\;$= 0.005 to reflect a realistic network density, with computations distributed across 12 CPU cores. Additional details on pre-processing, as well as results pertaining to network sizes, sample sizes, and sparsity levels, are provided in Supplementary Section S4.

We first compared the efficiency of iDDN, DDN, and JGL on a two-layer network using the RNA-seq data. iDINGO was excluded from this comparison as it has been shown to be significantly slower in prior studies (Fu *et al.* 2024). As reported in Supplementary Table S1, JGL was efficient for small networks with fewer than 100 genes. However, for networks containing 1000 genes, JGL required over 45 min, whereas iDDN completed the task in just 7.2 s. This demonstrates that iDDN is substantially more efficient than JGL, even for networks of moderate size. For smaller networks, iDDN showed no major performance advantage over DDN due to the relatively high overhead cost of initializing parallel computing. However, this overhead becomes negligible for larger networks (as detailed in Supplementary Table S2). Next, we assessed the performance of iDDN on a large three-layer network composed of TFs, ATACs, and non-TF mRNAs (Supplementary Section S8.2). In this network, TFs were allowed to connect to any ATAC site, while each ATAC site could only connect to mRNAs located on the same chromosome. After merging the RNA and ATAC datasets, the final dataset comprised 1344 T cells, 1098 monocytes, 19 131 mRNAs (including 1163 TFs), and 90 451 ATAC sites. We tested the running time and memory usage of iDDN on this large network under different sparsity penalties (Table 1). iDDN completed each task in approximately 30 min, requiring around 20 GB of memory, including the memory used for loading datasets. These results demonstrate that iDDN is not only more efficient than peer methods but is also well-suited for application on large-scale datasets.

**Table 1.** Running time and memory usage of iDDN on a large network with 109582 features and 2442 samples based on PBMC data.

$\lambda_{1}$	Time (s)	Memory (GB)	Network density
0.025	1718	20.6	0.1185
0.05	1669	19.4	0.0299
0.1	1662	19.1	0.0046

## 4 Biological case studies

We first applied iDDN to three in-house multi-omics datasets. These datasets were chosen because they represent high-quality multi-omics data acquired from the same subjects, addressing scientific questions related to diseases. In addition, we applied iDDN to a publicly available PBMC single-cell multi-omics dataset. Here, we present the applications on the CPTAC and GPAA datasets. The results for the other two datasets are available in Supplementary Sections S7 and S8, respectively.

### 4.1 CPTAC ovarian cancer data

We applied iDDN to multi-omics data from the CPTAC-OV2 prospective study, which performed a comprehensive proteomics and genomics characterization of human ovarian high-grade serous carcinoma tumors (Zhang *et al.* 2016). For 82 ovarian cancer tumor samples, we obtained matching RNA-seq data from the NCI database. After performing total count normalization, we excluded features with more than 10% missing data and applied mean imputation. Among these samples, 19 with somatic mutations in the BRCA1, BRCA2 or PTEN genes were categorized as homologous recombination deficiency (HRD+), while 63 were grouped as HRD−. From a list of 171 HRD-associated genes, we identified 120 that were present in the RNA-seq data. Using the TRRUST database (Han *et al.* 2018), we identified the top up-regulating transcription factors (TFs) for these genes, uncovering 53 TFs with at least three regulated target genes (*P*-value < 0.005, FDR < 0.01). Of these, 23 TFs were also available in the proteomics data. The processed dataset included the expression of 120 mRNAs and 23 TF proteins. TF-binding information was incorporated as constraints by iDDN during network inference. Using 5-fold cross-validation and the one-standard-error rule, we selected $\lambda_{1}=0.131$ for this study.

**Figure 3.**
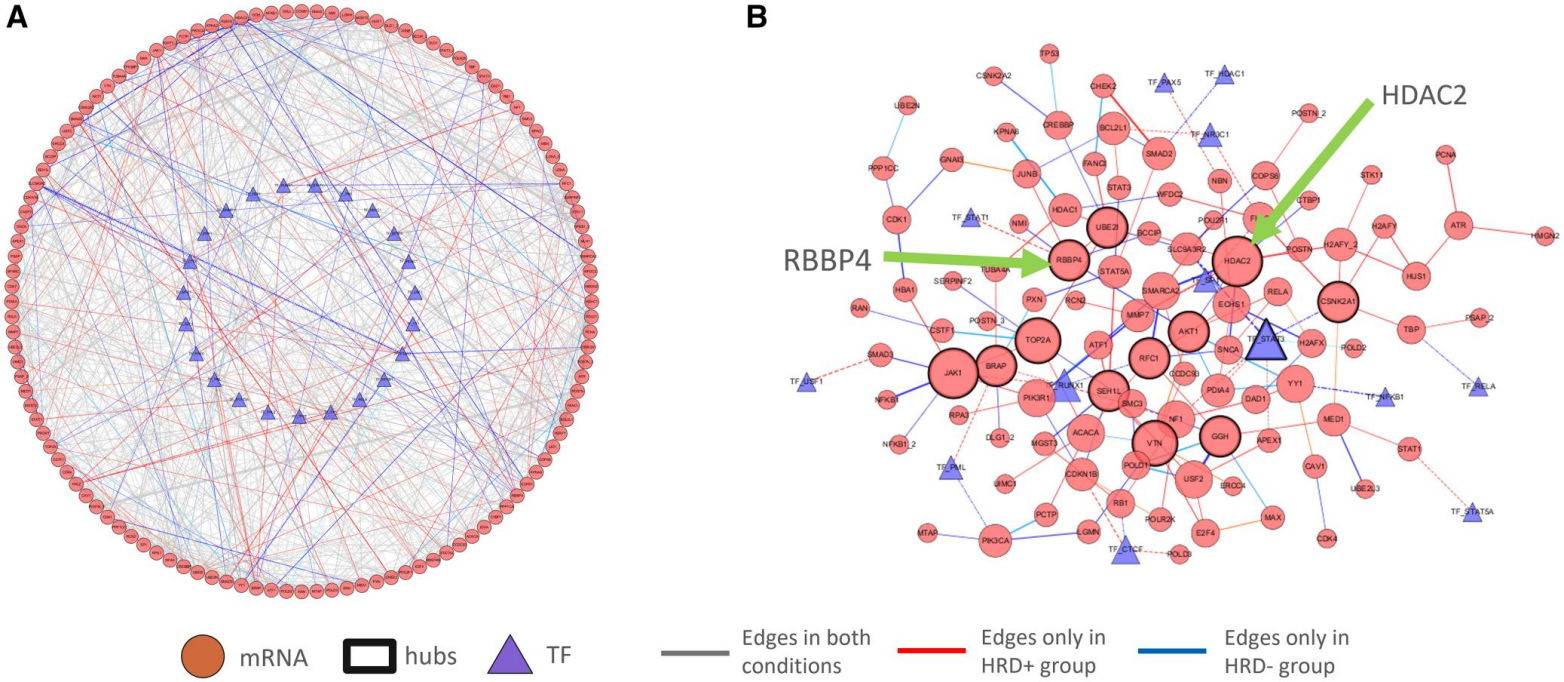
Application of iDDN on CPTAC ovarian cancer data with HRD+ and HRD− samples. (A) The common and differential networks inferred by iDDN. Common edges (static dependencies), as well as differential edges for each HRD group, are depicted. TFs are shown as triangles, and target mRNAs are displayed as circles. The thickness of each edge corresponds to the weight of the connection. (B) A focused subset of (A) highlighting biologically significant network rewiring. Hub nodes are marked with thick outlines. Two key genes, HDAC2 and RBBP4, are emphasized with arrows.

Figure 3A shows the reconstructed common and differential networks. iDDN detected 632 common intra-omics connections among the 120 genes and 107 differential intra-omics connections. For inter-omics links between TF and RNA nodes, 68 common edges and 25 differential edges were identified. Figure 3B highlights the rewiring detected in the differential network, where 11 genes and one TF were identified as hubs with high connectivity degrees. Among these hubs, RBBP4 is one of the five genes (HDAC1, RBBP4, RBBP7, EP300, and HUS1) involved in histone acetylation or deacetylation, as previously identified by the CPTAC-OV1 project (Zhang *et al.* 2016). Additionally, the hub gene HDAC2 was found to be a histone deacetylase.

### 4.2 GPAA human artery data

**Figure 4.**
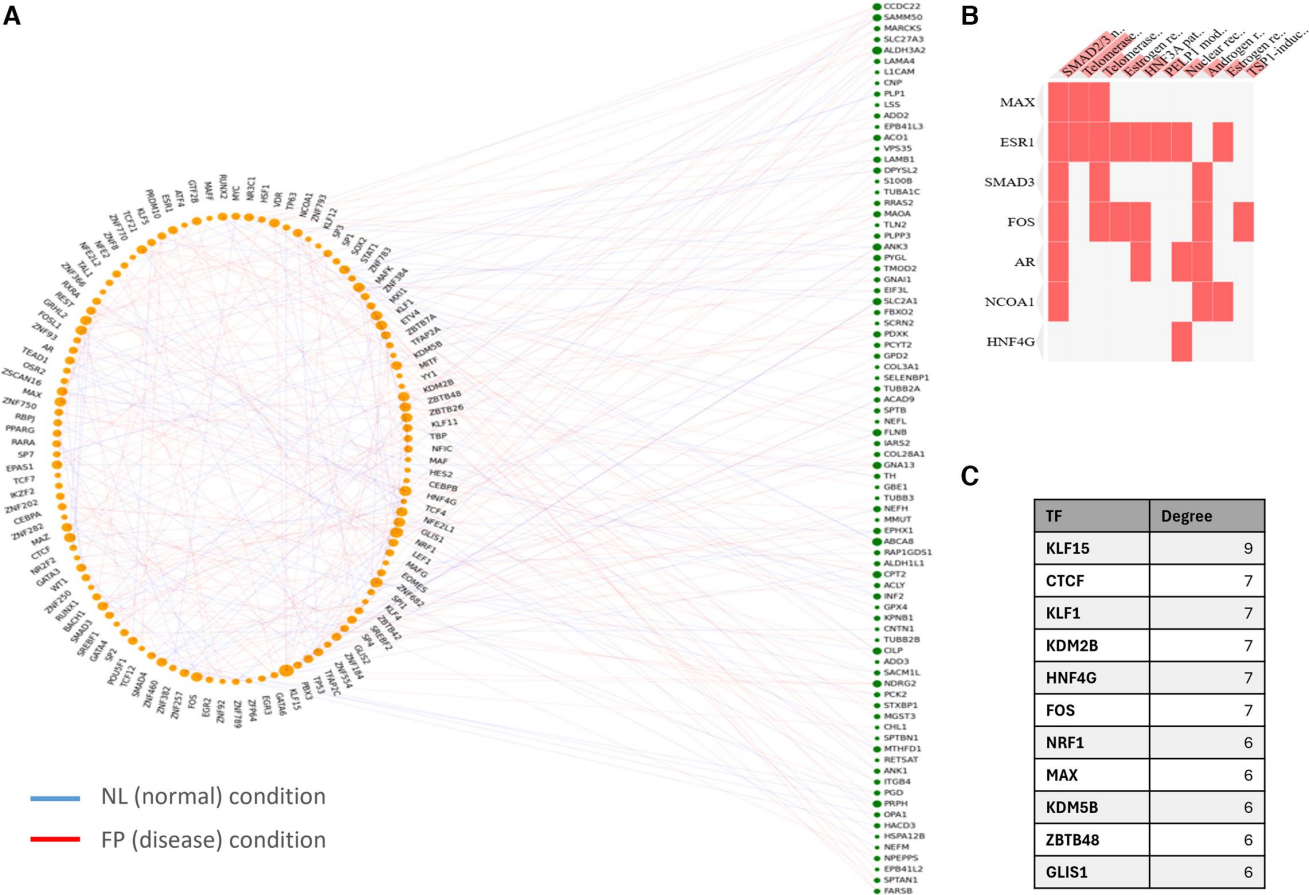
Application of iDDN on GPAA coronary artery RNA-seq and proteomics data. (A) Differential network estimated by iDDN. TFs are shown on the left, while signature gene mRNAs are on the right. The size of each node corresponds to its degree. Lines with different colors are used to represent edges only in the NL (normal) condition or edges only in the FP (disease) condition. (B) Cluster-gram linking hub TFs in the differential network to their corresponding enriched pathways, based on the BioPlanet 2019 database in Enrichr. Columns represent enriched terms, and rows correspond to input genes. Each matrix element indicates whether a gene is associated with a specific term. (C) Top hub TFs in the differential network derived from GPAA data. Only TFs with at least six rewired edges between the two conditions are shown.

We applied iDDN to the GPAA human coronary artery data (Herrington *et al.* 2018), which includes matched mRNA and proteomics data for the same subject. Using the CAM3.0 method (Wu *et al.* 2024) on the proteomics data, we identified seven latent features (LF) corresponding to biological processes that contribute to atherosclerosis progression. For each LF, we determined the top 100 signature genes (SG) that are exclusively highly expressed. For each LF, we are interested in identifying TFs that regulate the expression of a significant portion of the SGs, as well as TFs whose regulatory effects significantly change between normal and disease conditions. Therefore, we applied iDDN to construct common and differential regulatory networks and identified hub TFs in each network. $\lambda_{1}$ and $\lambda_{2}$ were selected to be 0.1 and 0.04, respectively, as these values provided reasonable numbers of hubs in common and differential networks. The differential network is illustrated in Fig. 4A, and hubs were defined as TFs with degrees ≥5 (Fig. 4C). Details regarding the common network results, feature filtering, constraints, and sample grouping are provided in Supplementary Section S6.

Pathway analysis was performed using the Enrichr web interface (Kuleshov *et al.* 2016), revealing several significant pathways associated with hub TFs in the common network. For example, the TNF-alpha signaling pathway had a *P*-value of 4.4e−8 in the MSigDB Hallmark 2022 database (Liberzon *et al.* 2015). Another significant pathway, the Transcriptional Cascade Regulating Adipogenesis, had a *P*-value of 6.3e−10 based on the WikiPathway 2023 Human database (Kutmon *et al.* 2016). Both pathways are strongly linked to Arteriosclerosis (Fantuzzi and Mazzone 2007, Kleinbongard *et al.* 2010).

From the differential network hub nodes, we also identified several pathways with significant *P*-values (Fig. 4B). For example, the SMAD2/3 pathway was highly significant, with a *P*-value of 1.4e−10 according to BioPlanet 2019 database (Huang *et al.* 2019). SMAD2 and SMAD3 are canonical downstream TFs in the transforming growth factor beta (TGFB) signaling pathway, which is implicated in atherosclerosis (Kalinina *et al.* 2004, Deng *et al.* 2021) and is known to have both protective and detrimental effects (Toma and McCaffrey 2012). Interestingly, FOS was found among the hub nodes. FOS is a signaling co-factor for SMADs and modulates the transcriptional response to TGFB (Zhang *et al.* 1998). Its identification as a hub in the differential network suggests a potentially detrimental shift in TGFB signaling characteristics.

## 5 Discussion

In this paper, we introduce iDDN as an accurate, efficient, and versatile tool for multi-omics differential network analysis. Built on a fused Lasso regression framework, iDDN enables biologically principled data integration with easy incorporation of *a priori* regulator-effector constraints. We demonstrate that iDDN outperforms related methods in both accuracy and efficiency. Additionally, we showcase its broad applicability through four real-world applications on diverse types of omics datasets. We believe iDDN offers new possibilities for understanding disease progression by inferring intra- and inter-omics network structures and detecting regulatory rewiring. In this section, we discuss the novelties of iDDN, potential challenges, and directions for future research. Further discussions can be found in Supplementary Section S9.

The methodological novelties of iDDN include: (i) a flexible layer-wise Gaussian Graphical Model (GGM) framework for integrating multi-omics data, (ii) the just-right incorporation of biologically plausible prior constraints, and (iii) highly effective acceleration strategies and implementations. As the result of these new features, iDDN outperforms closely related peer methods, including JGL (Danaher *et al.* 2014), iDINGO (Class *et al.* 2018), and DDN3.0 (Fu *et al.* 2024), in terms of accuracy, efficiency, and applicability. Furthermore, iDDN is able to jointly model networks under two conditions with any number of omics types. These novel functions make iDDN potentially superior to existing GRN inference methods (Huynh-Thu *et al.* 2010, Yuan and Duren 2025) and generic network inference approaches (Langfelder and Horvath 2008). Additional discussion can be found in Supplementary Section S9.1.

One challenge of iDDN is modeling nonlinear effects. While methods based on nonlinear models may perform better for some networks, iDDN is designed to complement, rather than replace, existing tools. It is worth emphasizing, however, that while iDDN adopts linear models for each condition, the combined common and rewired networks inferred by iDDN collectively capture a biologically plausible and significant form of nonlinear interactions across different biological conditions (Manicka *et al.* 2023, Kadelka and Murrugarra 2024) (Supplementary Section S9.3). Another challenge lies in handling noisy data. Alongside quality control and preprocessing (Zhang *et al.* 2016), iDDN leverages biologically plausible constraints to help mitigate spurious signals. For datasets with a high rate of missing values, we recommend first removing features or samples with very high levels of missing data, followed by a tailored application of established imputation methods (Shen *et al.* 2022, Du *et al.* 2024) (Supplementary Section S9.4).

Our future work will focus on three key aspects: (i) As highlighted in GRN benchmark papers, different methods may be better suited for specific networks and omics types. To this end, we plan to comprehensively evaluate the accuracy of iDDN using various benchmark datasets and compare its performance with existing GRN inference tools, such as GENIE3 (Huynh-Thu *et al.* 2010), SCENIC+ (Bravo González-Blas *et al.* 2023), and LINGER (Yuan and Duren 2025). (ii) We will adapt and extend iDDN to address critical multi-omics network inference tasks, leveraging the unique properties of relevant datasets. For example, we aim to explore the integration of ATAC-seq data to enhance network inference and provide additional biological insights. (iii) We will identify causal regulators using *in silico* perturbation approaches. For instance, we may combinatorially reestablish the wiring of a normal network from a disease-altered network to detect critical rewiring events, along with the associated nodes and edges (Kamimoto *et al.* 2023). Alternatively, we may use the regulatory impact factor score for each node (Reverter *et al.* 2010) to identify significant regulators.

## Supplementary Material

vbaf086_Supplementary_Data

## Data Availability

The synthetic data, CPTAC data, and kinase data used in this work are available on Zenodo (https://zenodo.org/records/14941037). The code for generating the synthetic data is available at https://github.com/cbil-vt/iddn_experiments. The CPTAC and kinase data are also available at https://pdc.cancer.gov/pdc/. The PBMC data is available at https://support.10xgenomics.com/single-cell-multiome-atac-gex/datasets/1.0.0/pbmc_granulocyte_sorted_10k. The GPAA data is available upon request.

## References

[vbaf086-B1] 10x Genomcis. PBMC from a healthy donor—granulocytes removed through cell sorting (10k). 2010. https://support.10xgenomics.com/single-cell-multiome-atac-gex/datasets/1.0.0/pbmc_granulocyte_sorted_10k

[vbaf086-B2] Bravo González-Blas C , De Winter S, Hulselmans G et al SCENIC+: single-cell multiomic inference of enhancers and gene regulatory networks. Nat Methods 2023;20:1355–67. 37443338 10.1038/s41592-023-01938-4 PMC10482700

[vbaf086-B3] Class CA , Ha MJ, Baladandayuthapani V et al iDINGO-integrative differential network analysis in genomics with shiny application. Bioinformatics 2018;34:1243–5. 29194470 10.1093/bioinformatics/btx750 PMC6030922

[vbaf086-B4] Cui H , Wang C, Maan H et al scGPT: toward building a foundation model for single-cell multi-omics using generative AI. Nat Methods 2024;21:1470–80. 38409223 10.1038/s41592-024-02201-0

[vbaf086-B5] Danaher P , Wang P, Witten DM. The joint graphical lasso for inverse covariance estimation across multiple classes. J R Stat Soc Series B Stat Methodol 2014;76:373–97. 24817823 10.1111/rssb.12033 PMC4012833

[vbaf086-B6] Deng H , Min E, Baeyens N et al Activation of Smad2/3 signaling by low fluid shear stress mediates artery inward remodeling. Proc Natl Acad Sci USA 2021;118:e2105339118. 10.1073/pnas.2105339118 PMC8449390 34504019

[vbaf086-B7] Du D , Bhardwaj S, Lu Y et al Embracing the informative missingness and silent gene in analyzing biologically diverse samples. Sci Rep 2024;14:28265. 39550430 10.1038/s41598-024-78076-0 PMC11569126

[vbaf086-B8] Fantuzzi G , Mazzone T. Adipose tissue and atherosclerosis: exploring the connection. Arterioscler Thromb Vasc Biol 2007;27:996–1003. 17303782 10.1161/ATVBAHA.106.131755

[vbaf086-B9] Friedman J , Hastie T, Tibshirani R. *The Elements of Statistical Learning*. New York: Springer Series in Statistics, 2017.

[vbaf086-B10] Fu Y , Lu Y, Wang Y et al DDN3.0: determining significant rewiring of biological network structure with differential dependency networks. Bioinformatics 2024;40:btae376. 10.1093/bioinformatics/btae376 PMC11199198 38902940

[vbaf086-B111] Han H , Cho J-W, Lee S et al TRRUST v2: an expanded reference database of human and mouse transcriptional regulatory interactions. Nucleic Acids Res 2018;46:380–86. 10.1093/nar/gkx1013 PMC5753191 29087512

[vbaf086-B11] Herrington DM , Mao C, Parker SJ et al Proteomic architecture of human coronary and aortic atherosclerosis. Circulation 2018;137:2741–56. 29915101 10.1161/CIRCULATIONAHA.118.034365 PMC6011234

[vbaf086-B12] Hu JX , Thomas CE, Brunak S. Network biology concepts in complex disease comorbidities. Nat Rev Genet 2016;17:615–29. 27498692 10.1038/nrg.2016.87

[vbaf086-B13] Huang R , Grishagin I, Wang Y et al The NCATS BioPlanet—an integrated platform for exploring the universe of cellular signaling pathways for toxicology, systems biology, and chemical genomics. Front Pharmacol 2019;10:445. 31133849 10.3389/fphar.2019.00445 PMC6524730

[vbaf086-B14] Huynh-Thu VA , Irrthum A, Wehenkel L et al Inferring regulatory networks from expression data using tree-based methods. PLoS One 2010;5:e12776. 10.1371/journal.pone.0012776 PMC2946910 20927193

[vbaf086-B15] Kadelka C , Murrugarra D. Canalization reduces the nonlinearity of regulation in biological networks. NPJ Syst Biol Appl 2024;10:67. 38871768 10.1038/s41540-024-00392-y PMC11176187

[vbaf086-B16] Kalinina N , Agrotis A, Antropova Y et al Smad expression in human atherosclerotic lesions: evidence for impaired TGF-beta/Smad signaling in smooth muscle cells of fibrofatty lesions. Arterioscler Thromb Vasc Biol 2004;24:1391–6. 15166010 10.1161/01.ATV.0000133605.89421.79

[vbaf086-B17] Kamimoto K , Stringa B, Hoffmann CM et al Dissecting cell identity via network inference and in silico gene perturbation. Nature 2023;614:742–51. 36755098 10.1038/s41586-022-05688-9 PMC9946838

[vbaf086-B18] Kleinbongard P , Heusch G, Schulz R. TNFalpha in atherosclerosis, myocardial ischemia/reperfusion and heart failure. Pharmacol Ther 2010;127:295–314. 20621692 10.1016/j.pharmthera.2010.05.002

[vbaf086-B19] Kuleshov MV , Jones MR, Rouillard AD et al Enrichr: a comprehensive gene set enrichment analysis web server 2016 update. Nucleic Acids Res 2016;44:W90–7. 27141961 10.1093/nar/gkw377 PMC4987924

[vbaf086-B20] Kutmon M , Riutta A, Nunes N et al WikiPathways: capturing the full diversity of pathway knowledge. Nucleic Acids Res 2016;44:D488–94. 26481357 10.1093/nar/gkv1024 PMC4702772

[vbaf086-B21] Langfelder P , Horvath S. WGCNA: an R package for weighted correlation network analysis. BMC Bioinformatics 2008;9:559. 19114008 10.1186/1471-2105-9-559 PMC2631488

[vbaf086-B22] Liberzon A , Birger C, Thorvaldsdóttir H et al The molecular signatures database (MSigDB) hallmark gene set collection. Cell Syst 2015;1:417–25. 26771021 10.1016/j.cels.2015.12.004 PMC4707969

[vbaf086-B23] Manicka S , Johnson K, Levin M et al The nonlinearity of regulation in biological networks. NPJ Syst Biol Appl 2023;9:10. 37015937 10.1038/s41540-023-00273-w PMC10073134

[vbaf086-B24] Meinshausen N , Bühlmann P. High-dimensional graphs and variable selection with the Lasso. Ann Stat 2006;34:1436–62.

[vbaf086-B25] Mitra K , Carvunis A-R, Ramesh SK et al Integrative approaches for finding modular structure in biological networks. Nat Rev Genet 2013;14:719–32. 24045689 10.1038/nrg3552 PMC3940161

[vbaf086-B26] Nouri N , Gaglia G, Mattoo H et al GENIX enables comparative network analysis of single-cell RNA sequencing to reveal signatures of therapeutic interventions. Cell Rep Methods 2024;4:100794. 38861988 10.1016/j.crmeth.2024.100794 PMC11228368

[vbaf086-B27] Pedregosa F , Varoquaux G, Gramfort A et al Scikit-learn: machine learning in Python. J Mach Learn Res 2011;12:2825–30.

[vbaf086-B28] Reverter A , Hudson NJ, Nagaraj SH et al Regulatory impact factors: unraveling the transcriptional regulation of complex traits from expression data. Bioinformatics 2010;26:896–904. 20144946 10.1093/bioinformatics/btq051

[vbaf086-B29] Shen M , Chang Y-T, Wu C-T et al Comparative assessment and novel strategy on methods for imputing proteomics data. Sci Rep 2022;12:1067. 35058491 10.1038/s41598-022-04938-0 PMC8776850

[vbaf086-B30] Theodoris CV , Xiao L, Chopra A et al Transfer learning enables predictions in network biology. Nature 2023;618:616–24. 37258680 10.1038/s41586-023-06139-9 PMC10949956

[vbaf086-B31] Tian Y , Zhang B, Hoffman EP et al Knowledge-fused differential dependency network models for detecting significant rewiring in biological networks. BMC Syst Biol 2014;8:87. 25055984 10.1186/s12918-014-0087-1 PMC4131167

[vbaf086-B32] Toma I , McCaffrey TA. Transforming growth factor-beta and atherosclerosis: interwoven atherogenic and atheroprotective aspects. Cell Tissue Res 2012;347:155–75. 21626289 10.1007/s00441-011-1189-3 PMC4915479

[vbaf086-B33] Wu C-T , Du D, Chen L et al CAM3.0: determining cell type composition and expression from bulk tissues with fully unsupervised deconvolution. Bioinformatics 2024;40:btae107. 10.1093/bioinformatics/btae107 PMC10924278 38407991

[vbaf086-B34] Yuan Q , Duren Z. Inferring gene regulatory networks from single-cell multiome data using atlas-scale external data. Nat Biotechnol 2025;43:247–57. 38609714 10.1038/s41587-024-02182-7 PMC11825371

[vbaf086-B35] Zhang B , Li H, Riggins RB et al Differential dependency network analysis to identify condition-specific topological changes in biological networks. Bioinformatics 2009;25:526–32. 19112081 10.1093/bioinformatics/btn660 PMC2642641

[vbaf086-B36] Zhang B , Wang Y. Learning structural changes of Gaussian graphical models in controlled experiments. In: *Proceedings of the 26th Conference on Uncertainty in Artificial Intelligence (UAI 2010)*, *Catalina Island, CA, USA*. Arlington, VA, USA: AUAI Press, 2010.

[vbaf086-B37] Zhang H , Liu T, Zhang Z et al, CPTAC Investigators. Integrated proteogenomic characterization of human high-grade serous ovarian cancer. Cell 2016;166:755–65. 27372738 10.1016/j.cell.2016.05.069 PMC4967013

[vbaf086-B38] Zhang Y , Feng XH, Derynck R. Smad3 and Smad4 cooperate with c-jun/c-fos to mediate TGF-beta-induced transcription. Nature 1998;394:909–13. 9732876 10.1038/29814

