# Supplementary material for: iDDN: determining trans-omics network structure and rewiring with integrative differential dependency networks
Source: Bioinform Adv. 2025 May 8;5(1):vbaf086. doi: 10.1093/bioadv/vbaf086 (PMC12070392; doi:10.1093/bioadv/vbaf086)
Supplement: vbaf086_Supplementary_Data [file vbaf086_supplementary_data.zip › iDDN SI 04 07 25.pdf]

Supplementary Information

iDDN: Determining trans-omics network structure and rewiring with integrative differential dependency networks

Yizhi Wang, Yi Fu, Yingzhou Lu, Zhen Zhang, Robert Clarke, Sarah J. Parker, David M. Herrington, Guoqiang Yu, and Yue Wang

Contents

1 Additional descriptions about methods 2
1.1 Solutions of BCD algorithm 2
1.2 Adaptation of residual updating acceleration strategy 3
1.3 Hyperparameter settings based on significant test 4
1.4 Implementation of Python package 5
1.5 Additional remarks on the methods 6
2 Simulation setup 7
2.1 Generation of synthetic network structures 7
2.2 Generation of synthetic multi-omics data 8
2.3 Performance evaluation 9
3 Evaluation of network inference accuracy 10
3.1 iDDN/DDN performance with varying number of layers 10
3.2 iDDN/DDN performance under different levels of constraints 13
3.3 Simulation setup for peer methods 16
3.4 Comparative evaluation with peer methods 17
4 Comparative evaluation of computational efficiency 20
5 Ablation studies of running time and inference accuracy 23
6 Analysis of GPAA human arterial data 25
7 Analysis of phosphorylation data from CPTAC 28
8 Analysis of single-cell PBMC data 30
8.1 Evaluation of iDDN cell-type specific GRN inference 30
8.2 Case study: three-layer TF-ATAC-mRNA network 32
9 Additional discussions 34
9.1 Novelties of iDDN and relationship with peer methods. 34
9.2 Our preference for moderately sized multi-omics datasets 35
9.3 Realities in modeling non-linear effects 36
9.4 Considerations with data with high noise and/or missing rate 36
9.5 Considerations on working on specific omics types and tasks. 37
References 38

1 Additional descriptions about methods

1.1 Solutions of BCD algorithm

For each node i in a graph with p nodes, iDDN determines the weights between nodes $\{1,2,\ldots,i-1,i+1,\ldots,p\}$ and node i under each condition, incorporating sparsity and similarity penalties. This task is efficiently performed using the BCD algorithm. During each step of BCD, we update the weights between node k to node i under each condition. We denote the weights as $\beta_k = (\beta_{k,1}, \beta_{k,2})$, the sparsity penalty between k and i as Λ_1, and the similarity penalty as Λ_2. Note that in iDDN these penalties can differ in different edges. We define $\rho^{(c)} = \left(\boldsymbol{y}_{\boldsymbol{i,resi}}^{(\boldsymbol{c})}\right)^T \boldsymbol{X}_{\boldsymbol{k}}^{(\boldsymbol{c})}$, where $\boldsymbol{X}_{\boldsymbol{k}}^{(\boldsymbol{c})}$ (shape $p \times 1$) is the k_{th} variable (column) of the input data, and $\boldsymbol{y}_{\boldsymbol{i,resi}}^{(\boldsymbol{c})}$ (shape $p \times 1$) is the residual signal $\boldsymbol{y}_{\boldsymbol{i,resi}}^{(\boldsymbol{c})} = \boldsymbol{y}_{\boldsymbol{i}}^{(\boldsymbol{c})} - \boldsymbol{X}_{A(i)\backslash j}^{(\boldsymbol{c})} \boldsymbol{\beta}_{i,A(i)\backslash j}^{(c)}$, as defined in Section 2.4 of the main text. c is 1 or 2, depending on the conditions We obtain closed-form solutions for β_k based on the relationship between $\rho^{(1)}$, $\rho^{(2)}$, Λ_1, and Λ_2, using the same derivation as (Zhang and Wang, 2010).

For $\rho^{(1)} \geq \rho^{(2)} + 2\Lambda_2, \rho^{(2)} \geq \Lambda_1 - \Lambda_2$,

$$\beta_k = \left(\rho^{(1)} - \Lambda_1 - \Lambda_2, \rho^{(2)} - \Lambda_1 + \Lambda_2\right)$$

For $\rho^{(1)} \leq \rho^{(2)} - 2\Lambda_2, \rho^{(2)} \leq -(\Lambda_1 - \Lambda_2)$,

$$\beta_k = \left(\rho^{(1)} + \Lambda_1 + \Lambda_2, \rho^{(2)} + \Lambda_1 - \Lambda_2\right)$$

For $\rho^{(1)} \geq \Lambda_1 - \Lambda_2, \rho^{(2)} \geq \rho^{(1)} + 2\Lambda_2$,

$$\beta_k = \left(\rho^{(1)} - \Lambda_1 + \Lambda_2, \rho^{(2)} - \Lambda_1 - \Lambda_2\right)$$

For $\rho^{(1)} \leq -(\Lambda_1 - \Lambda_2), \rho^{(2)} \leq \rho^{(1)} - 2\Lambda_2$,

$$\beta_k = \left(\rho^{(1)} + \Lambda_1 - \Lambda_2, \rho^{(2)} + \Lambda_1 + \Lambda_2\right)$$

For $\rho^{(1)} < \rho^{(2)} + 2\Lambda_2, \rho^{(2)} < \rho^{(1)} + 2\Lambda_2, \rho^{(2)} > -\rho^{(1)} + 2\Lambda_1$,

$$\beta_k = \left(\frac{1}{2}\left(\rho^{(1)} + \rho^{(2)}\right) - \Lambda_1, \frac{1}{2}\left(\rho^{(1)} + \rho^{(2)}\right) - \Lambda_1\right)$$

For $\rho^{(1)} > \rho^{(2)} - 2\Lambda_2, \rho^{(2)} > \rho^{(1)} - 2\Lambda_2, \rho^{(2)} < -\rho^{(1)} - 2\Lambda_1$,

$$\beta_k = \left(\frac{1}{2}\left(\rho^{(1)} + \rho^{(2)}\right) + \Lambda_1, \frac{1}{2}\left(\rho^{(1)} + \rho^{(2)}\right) + \Lambda_1\right)$$

For $\rho^{(1)} < \Lambda_1 - \Lambda_2, \rho^{(1)} > -\Lambda_1 - \Lambda_2, \rho^{(2)} > \Lambda_1 + \Lambda_2$

$$\beta_k = \left(0, \rho^{(2)} - \Lambda_1 - \Lambda_2\right)$$

For $\rho^{(1)} > -\Lambda_1 + \Lambda_2, \rho^{(1)} < \Lambda_1 + \Lambda_2, \rho^{(2)} < -\Lambda_1 - \Lambda_2$

$$\beta_k = \left(0, \rho^{(2)} + \Lambda_1 + \Lambda_2\right)$$

For $\rho^{(1)} > \Lambda_1 + \Lambda_2, \rho^{(2)} > -\Lambda_1 - \Lambda_2, \rho^{(2)} < \Lambda_1 - \Lambda_2$

$$\beta_k = \left(\rho^{(1)} - \Lambda_1 - \Lambda_2, 0\right)$$

For $\rho^{(1)} < -\Lambda_1 - \Lambda_2, \rho^{(2)} < \Lambda_1 + \Lambda_2, \rho^{(2)} > -\Lambda_1 + \Lambda_2$

$$\beta_k = \left(\rho^{(1)} + \Lambda_1 + \Lambda_2, 0\right)$$

For $\rho^{(1)} \geq \Lambda_1 + \Lambda_2, \rho^{(2)} \leq -\Lambda_1 - \Lambda_2$

$$\beta_k = \left(\rho^{(1)} - \Lambda_1 - \Lambda_2, \rho^{(2)} + \Lambda_1 + \Lambda_2\right)$$

For $\rho^{(1)} \leq -\Lambda_1 - \Lambda_2, \rho^{(2)} \geq \Lambda_1 + \Lambda_2$

$$\beta_k = \left(\rho^{(1)} + \Lambda_1 + \Lambda_2, \rho^{(2)} - \Lambda_1 - \Lambda_2\right)$$

For other cases,

$$\beta_k = (0,0)$$

In the next iteration, the BCD algorithm updates the weight between node $k + 1$ and node i. After it reaches node p, it will continue with node 1, until convergence is achieved. The whole process is then repeated to find weights between all nodes and node $i + 1$.

1.2 Adaptation of residual updating acceleration strategy

Several algorithmic acceleration strategies were developed in DDN3.0 (Fu, et al., 2024) to further improve the efficiency of the BCD algorithm. Among these, the residual update strategy is the most effective, as it scales well with a large number of features, a common scenario for multi-omics data. Another approach, the correlation matrix update strategy, is more efficient for very large sample sizes. However, we found that the residual update strategy is also highly efficient,

even for datasets with thousands of samples. Due to its broader applicability, we focus here on adapting the residual update strategy to the iDDN framework. Recall that for node i, when the BCD algorithm update node $j \in A(i)$, we need to calculate the residual signal using (potentially a large number of) nodes $A(i)\backslash j$ for each condition c:

$$\boldsymbol{y}_{\boldsymbol{i},\boldsymbol{resi}}^{(\boldsymbol{c})} = \boldsymbol{y}_{\boldsymbol{i}}^{(\boldsymbol{c})} - \boldsymbol{X}_{\boldsymbol{A}(\boldsymbol{i})\backslash \boldsymbol{j}}^{(\boldsymbol{c})} \boldsymbol{\beta}_{i,A(i)\backslash j}^{(c)}.$$

This step needs to be repeated when BCD works on another node in $A(i)$, which can be very time consuming when we have lots of nodes. However, it is clear that during two consecutive steps of BCD, most parts of $\boldsymbol{\beta}_{i,A(i)}^{(c)}$ is not changing at all. Therefore, instead of doing the matrix vector multiplication for all nodes in $A(i)$, after updating $\beta_{i,j}^{(1)}$ and $\beta_{i,j}^{(2)}$ in BCD, to work on $j+1$, we simply need to update the residual as follows:

$$\boldsymbol{y}_{\boldsymbol{i},\boldsymbol{resi}}^{(\boldsymbol{c})} = \boldsymbol{y}_{\boldsymbol{i},\boldsymbol{resi}}^{(\boldsymbol{c})} - \boldsymbol{X}_{\boldsymbol{j}}^{(\boldsymbol{c})} \boldsymbol{\beta}_{i,j}^{(c)} + \boldsymbol{X}_{\boldsymbol{j}+\boldsymbol{1}}^{(\boldsymbol{c})} \boldsymbol{\beta}_{i,j+1}^{(c)}.$$

Besides, we note that iDDN works on each node independently. In other words, solving objective function $L_{iddn}(i)$ is not influenced by $L_{iddn}(j)$. This independence enables the effective use of parallel computing in the iDDN implementation.

1.3 Hyperparameter settings based on significant test

Some general considerations for selecting hyperparameters in iDDN are discussed in the main text. Additionally, users are encouraged to consult the hyperparameter tuning tutorial in the iDDN documentation (https://iddn.readthedocs.io/). For small datasets, it is recommended to use a two-dimensional cross-validation grid search that jointly optimizes λ_1 and λ_2. For larger datasets, a sequential strategy may be adopted: first, use cross-validation to select λ_1, and then search for λ_2 while keeping λ_1 fixed. While this approach reduces computational resource requirements, it may slightly compromise accuracy. Below, we describe a purely statistical method for specifying the hyperparameter λ_2 using a given significance level. Although computationally simple, we generally recommend cross-validation-based approaches for better performance.

The following procedure is adapted from (Zhang and Wang, 2010). From the solution subregions in the plane of (ρ_1, ρ_2), we note that $\beta_k^{(1)}$and $\beta_k^{(2)}$ will be identical in the subregion of $|\rho_1 - \rho_2| < 2\lambda_2$. The key question is determining the value of $|\rho_1 - \rho_2|$ that is large enough for a

given significance level. We apply Fisher's transform to standardize correlation coefficients ρ_1 and ρ_2:

$$z_1 = \frac{1}{2} ln \frac{1+\rho_1}{1-\rho_1}, z_2 = \frac{1}{2} ln \frac{1+\rho_2}{1-\rho_2}.$$

Here z_1 and z_2 approximately follows $N\left(\frac{1}{2} ln \frac{1+\bar{\rho}_1}{1-\bar{\rho}_1}, \frac{1}{n_1-3}\right)$ and $N\left(\frac{1}{2} ln \frac{1+\bar{\rho}_2}{1-\bar{\rho}_2}, \frac{1}{n_2-3}\right)$. Since ρ_1 and ρ_2 are equal under the null hypothesis (no differential edges), z_1 and z_2 share the same mean. Assuming the independence between ρ_1 and ρ_2, $z = z_1 - z_2$ follows a normal distribution with zero-mean and variance $1/(n_1 - 3) + 1/(n_2 - 3)$. For a given significance level α (for example, 0.05), we define the significance threshold for $|z| = |z_1 - z_2|$ as:

$$s(\alpha) = \sqrt{\frac{1}{n_1 - 3} + \frac{1}{n_2 - 3}} \Phi^{-1}\left(1 - \frac{\alpha}{2}\right) < |z| = |z_1 - z_2|.$$

Rewriting the above equation in terms of ρ_1 and ρ_2, we have:

$$|z| = |z_1 - z_2| > s(\alpha) \Leftrightarrow |\rho_1 - \rho_2| > \frac{e^{2s(\alpha)} - 1}{e^{2s(\alpha)} + 1}(1 - \rho_1\rho_2) = 2\lambda_2$$

Since λ_2 is applied for all nodes' optimization, we replace $\rho_1\rho_2$ with the mean values estimated from all samples: $\rho_1\rho_2 \leftarrow \overline{\rho_1\rho_2}$. Finally, we get λ_2 under significance level α as

$$\lambda_2 = \frac{e^{2s(\alpha)} - 1}{2(e^{2s(\alpha)} + 1)}(1 - \overline{\rho_1\rho_2}).$$

1.4 Implementation of Python package

We implemented the iDDN algorithm as a Python package, enabling users to specify constraints among pairs of molecules through a dependency matrix (**Figure 1A** in the main text). Users also have precise control over the sparsity parameters for each edge. Core functions in iDDN were accelerated using Numba (https://numba.pydata.org/) (Lam, et al., 2015). Parallel computing was implemented with Joblib (https://joblib.readthedocs.io/), ensuring compatibility across multiple platforms. The source code is available at https://github.com/cbil-vt/iDDN and the package was published on PyPI (https://pypi.org/project/iddn/).

Additionally, we included several network visualization functions tailored for multi-omics data (Figure 1A in the main text, bottom). These functions are highly flexible, support any number of

layers, and are particularly well-suited for common and differential network analysis tasks. A set of tutorials and API references are also available (https://iddn.readthedocs.io/).

1.5 Additional remarks on the methods

The data types considered in iDDN may include, but are not limited to, mRNA, protein, miRNA, lncRNA, and metabolite. These can be organized into intra-omics layers, along with edges both within and between layers. For instance, since transcription factors (TFs) may exist in either protein or mRNA form, an intra-omics layer must accommodate both regulators and effectors—this flexibility is a key feature supported by iDDN. Without such constraints, conditional dependency-based models like DDN and JGL typically struggle to reliably infer true regulatory directions. By incorporating these constraints, iDDN can better prioritize the search space, reduce false positives, improve computational efficiency, and enhance interpretability.

Others and we have previously demonstrated the performance and utility of DDN on gene expression data (Tian, et al., 2014; Zhang, et al., 2009) and proteomics data (Herrington, et al., 2018; Zhang, et al., 2016). For readers interested in the mathematical formulation, algorithmic workflow, and comparative evaluations of DDN versus peer methods, we recommend the original reports (Fu, et al., 2024; Tian, et al., 2014) and comprehensive reviews (Hu, et al., 2016; Mitra, et al., 2013).

2 Simulation setup

2.1 Generation of synthetic network structures

Here, we introduce the general design of the synthetic networks used in simulation studies. It is worth noting that each simulation study may apply specific modifications to the network.

We designed networks with three layers: mRNA, TF protein, and miRNA (Figure S1), each containing 50 nodes. Edges were added within mRNAs, within TFs, between TFs and mRNAs, and between miRNAs and mRNAs. Two distinct scenarios were used to mimic varying regulatory impacts on the mRNAs of interest. In the first scenario, each regulator (TF or miRNA) regulated two mRNAs ($\delta = 2$); in the second scenario, each regulator could regulate five mRNAs ($\delta = 5$). The regulator layers and the mRNA layer were more tightly connected in the second scenario.

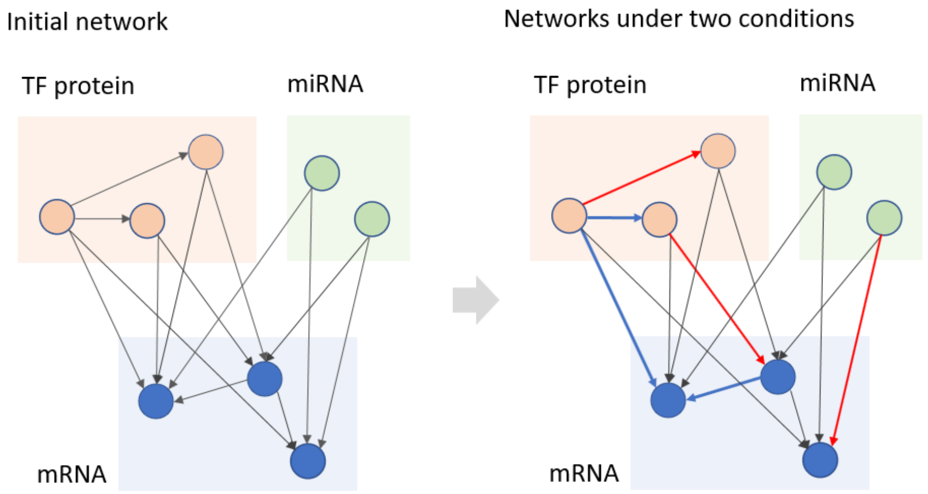

Figure S1. Illustration of network topology and the generation of two conditions. Left**:** A network with three layers, where the edges indicate regulatory relationships. Right**:** Edges specific to condition 1 (blue), edges specific to condition 2 (red), and edges present in both conditions (grey).

Nodes in the mRNA and TF layers were organized into two scale-free graphs. These graphs were generated using the generate function from the HUGE R package (Zhao, et al., 2012), which implements the B-A algorithm (Barabasi and Albert, 1999). For each TF or miRNA node, two mRNAs (first scenario) or five mRNAs (second scenario) were randomly assigned as targets. No interactions were assumed among miRNAs.

To create two conditions, 25% of the edges were randomly removed from the network under each condition, ensuring that different sets of edges were removed for each condition (Figure S1, right). The removed edges could be either within a layer or between layers. Using these two networks under the two conditions, we identified common edges (present in both conditions) and differential edges (present in only one condition), which served as the ground truth for our analysis.

2.2 Generation of synthetic multi-omics data

In this work, we created synthetic networks with varying numbers of layers and nodes. However, the steps for generating synthetic data from a given network remain consistent and are described below.

We began with two synthetic networks, one for each condition, and created an adjacency matrix A for each network. In these matrices, $A_{ij} = A_{ji} \neq 0$ if an edge existed between nodes i and j under that condition. Different weights were assigned to edges in the AA matrix: edges within each layer (e.g., TF-TF or mRNA-mRNA edges) were randomly assigned weights of 1 or -1, as were edges between TFs and mRNAs. Edges between miRNAs and mRNAs were assigned a weight of -1, reflecting their role in negative regulation. These assignments aimed to represent both positive and negative regulatory interactions.

The A matrix was not initially suitable for generating multivariate normal samples, as it might not have been positive definite. To resolve this, we employed a method similar to the approach used in the HUGE package (Zhao, et al., 2012). First, we calculated the eigenvalues of A and denoted the smallest one as e. Then we calculated $\Theta = 0.9A + w(|e| + 0.2)I$, where I is the identity matrix with the same shape as A, and w was set to 0.8 when $\delta = 2$ or 0.75 when $\delta = 5$, where δ is the number of edges each regulator connects. These adjustments ensured that Θ was positive definite, with the choice of w allowing for partial correlation tuning. The inverse of Θ was scaled to obtain a covariance matrix Ω. Specifically, let $B = \sqrt{\text{diag}(1/\Theta^{-1})}$, we got $\Sigma = B\Theta^{-1}B$, which transformed the diagonal elements of Θ^{-1} to 1 (Danaher, et al., 2014). We denoted covariance matrices under two conditions as Σ_1 and Σ_2. For each condition, we generated 200 samples using NumPy's "multivariate_normal" function (Harris, et al., 2020). This process was repeated 50 times; in each repetition, the network structure was regenerated, and weights were reassigned to avoid bias toward any specific set of edge connections or weights.

2.3 Performance evaluation

iDDN estimated the networks for each condition and represented the results as two coefficient matrices. Since the simulations focused solely on the existence of edges rather than their weights, we took the absolute value of each matrix and applied a small threshold (1×10^{-4}) to binarize the results. The two resulting matrices were used to calculate common edges (edges present in both conditions) and differential edges (edges present in only one condition).

Running iDDN required selecting two hyperparameters: λ_1 and λ_2, both of which could influence the results. To minimize the impact of hyperparameter selection in the simulations, we tested iDDN and peer methods across a wide range of parameter combinations. Specifically, λ_1 values ranged from 0.02 to 0.8 in increments of 0.02, while λ_2 values ranged from 0 to 0.15 in increments of 0.01. This resulted in 40 λ_1 values and 16 λ_2 values, yielding a total of 640 hyperparameter combinations.

The simulations were conducted using these 640 combinations of (λ_1, λ_2), repeated 30 times. To summarize the results and enable clearer visualization, for each λ_1, we selected the λ_2 that produced the best F1 score. Specifically, $\lambda_2^*(\lambda_1) = \text{argmin}_{\lambda_2} F_1(\lambda_1, \lambda_2)$, where F1 is the harmonic mean of precision and recall. In each case we plotted curves corresponding to different values of λ_1, where each point representing either the pROC or F1 score at hyperparameters $(\lambda_1, \lambda_2^*(\lambda_1))$.

3 Evaluation of network inference accuracy

3.1 iDDN/DDN performance with varying number of layers

We designed a simulation study to demonstrate that iDDN can achieve higher accuracy when inferring networks from data with multiple omics types. Below, we outline the motivation for our simulation design. Suppose we have selected a set of genes within a pathway and are interested in estimating their common and differential networks. Often, only mRNA data for these genes is available, allowing for the direct application of DDN3.0 to infer the networks. Now, consider the availability of additional data on potential regulators of these genes, such as transcription factor (TF) proteins and miRNAs. A natural question arises: does incorporating these extra datasets improve the estimation of gene networks? Specifically, we aim to determine whether the networks among mRNAs can be better inferred when additional regulatory layers are included in the signaling model. To evaluate the impact of incorporating additional regulatory layers, we provided iDDN with varying numbers of omics data types. Specifically, we generated four scenarios: 1) mRNA layer only, 2) mRNA + TF protein, 3) mRNA + miRNA, and 4) mRNA + TF protein + miRNA. For the first case, we simply applied DDN3.0. The evaluation focused solely on the mRNA layer, reflecting the study's primary motivation.

We first considered the scenario where each TF protein or miRNA was connected to two mRNAs, simulating a loosely connected relationship between the regulator and target layers (Figure S2). In the top left panel, the partial ROC (pROC) analysis compares the performance of the four combinations of omics types. Here, a smaller false positive rate (FPR) and a larger true positive rate (TPR) indicate better performance. Incorporating more omics types consistently improved performance in pROC analyses for estimating common network edges. In the top right panel, we evaluated the F1 score across various λ_1 values for common network estimation. In the bottom left panel, we analyzed the F1 score for differential network estimation. Once again, incorporating more omics types led to better accuracy. Interestingly, the benefits of including additional omics types were more pronounced in differential network estimation than in common network estimation. This is likely because inferring differential networks is inherently more challenging, and the availability of additional information has a more substantial impact. Finally, the bottom right panel presents the average F1 scores for both common and differential network estimations, further underscoring the advantages of incorporating multiple omics data types.

Next, we examined the scenario in which each TF protein or miRNA was connected to five mRNAs, representing a tighter connection between regulator layers and the mRNA layer (Figure S3). The top left panel presents the results of estimating the common network. Using multi-omics data improved pROC performance, and similar conclusions can be drawn from the F1 scores for the common networks shown in the top right panel. Results in the lower two panels demonstrate that the F1 scores for both the differential network and the overall performance were also enhanced. However, this simulation scenario was more challenging than the previous one, as the network was less sparse. Consequently, performance in this case was generally worse than in the earlier scenario.

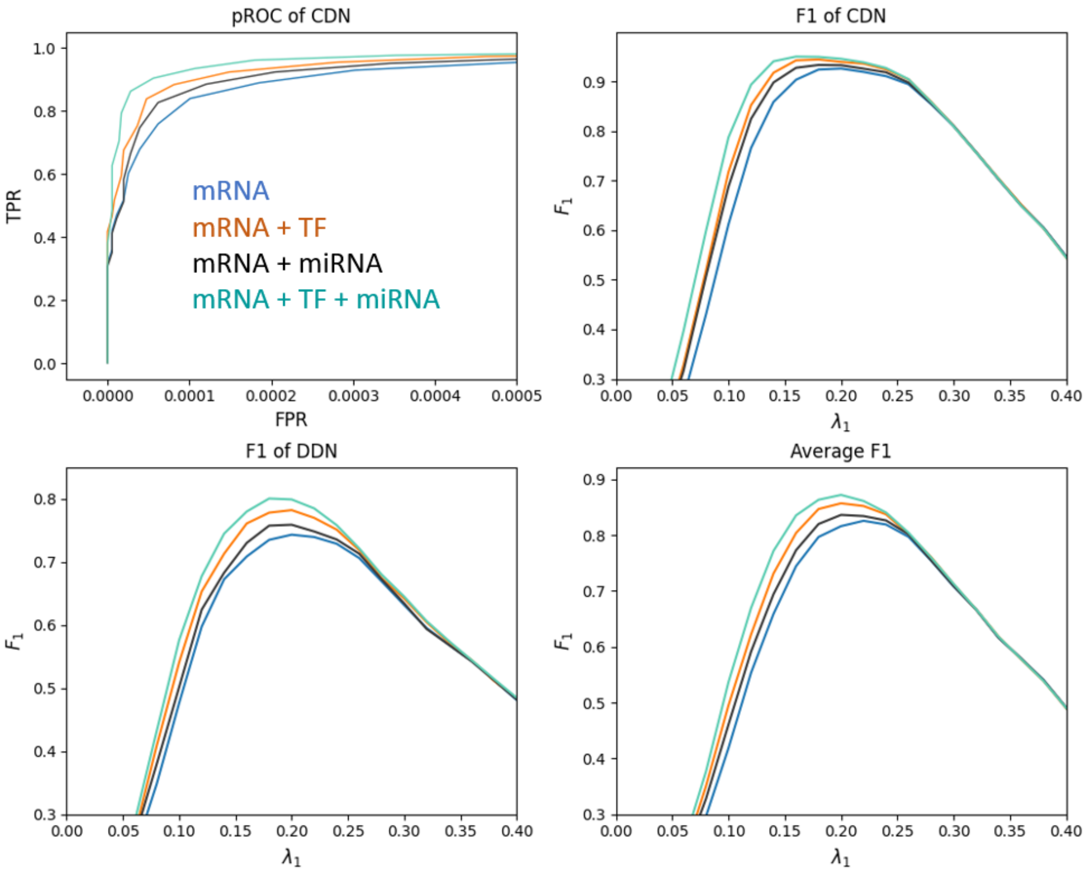

Figure S2. Performance comparison illustrating the benefits of integrating multi-omics data using iDDN. In this scenario, each TF node or miRNA node is connected to two mRNAs. The **top left panel** shows the partial ROC (pROC) performance for estimating the common network as λ_1 varies. The **top right panel** depicts the F1 score for the common network as λ_1 changes. The **bottom left panel** presents the F1 score for the differential network estimation, and the **bottom right panel** displays the average F1 score. For all panels, the results for each λ_1 are based on the best λ_2 value obtained from the scanned range.

The above experiments show that by incorporating multiple omics types, iDDN is able to improve the accuracy of the inference of both the common and the differential networks. Overall, when the number of edges between TF protein/miRNA and mRNA is small (e.g., two targets for each regulator), the benefits are smaller than when the connections are tighter between layers (e.g., five targets for each regulator). This result is expected because when different omics layers are tightly connected, ignoring one or more layers will more likely lead to inaccurate estimation of the edges in the remaining layers.

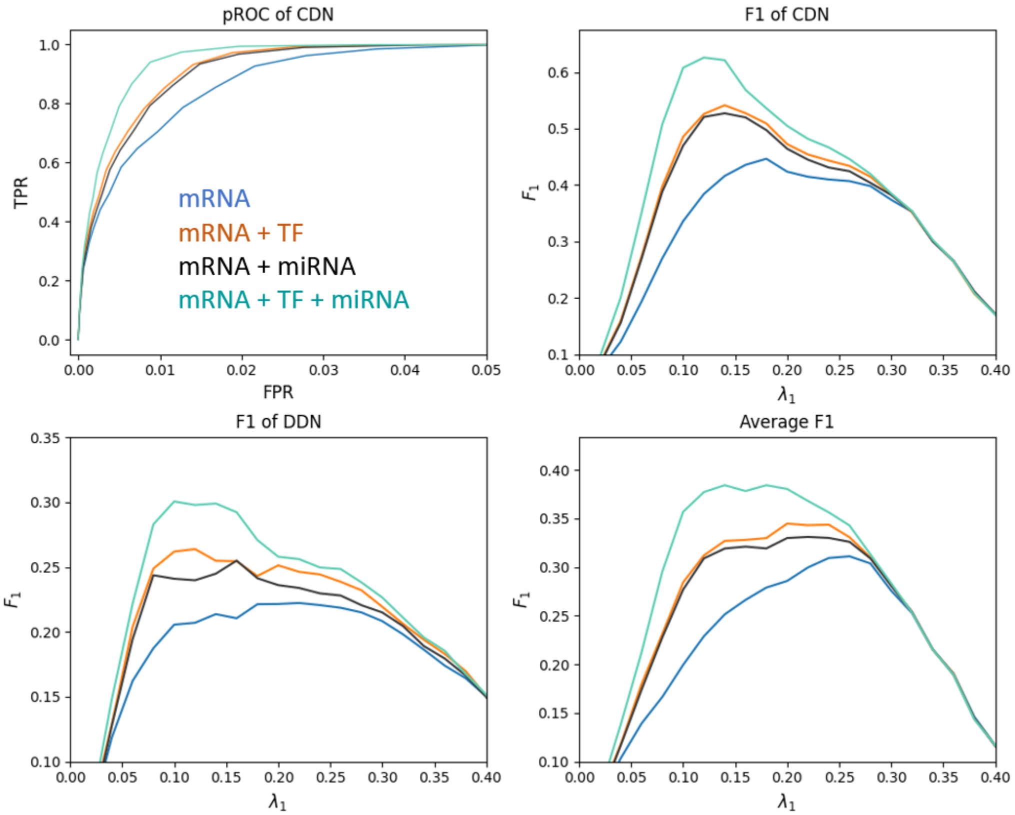

Figure S3. Performance comparison illustrating the benefits of integrating multi-omics data using iDDN. In this scenario, each TF node or miRNA node is connected to five mRNAs. The four panels were generated the same way as in Figure S2.

3.2 iDDN/DDN performance under different levels of constraints

In this section, we analyze the performance of iDDN under varying levels of constraints. We begin with some background information and a description of the constraints applied in the simulation, followed by an examination of their impact on iDDN's performance.

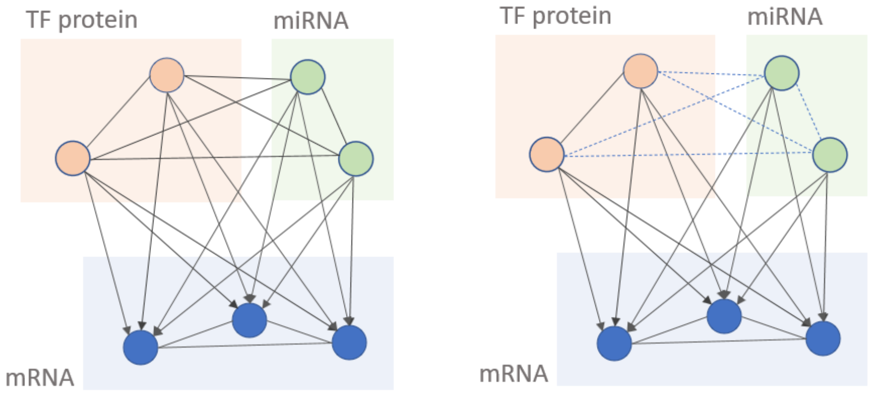

Figure S4. Illustrations of the incorporation of constraints in iDDN. Left: all edges are allowed in DDN3.0 and peer methods. Right: iDDN incorporates biological knowledge as regulatory constraints. Some edges are no longer allowed, which are shown as dashed lines.

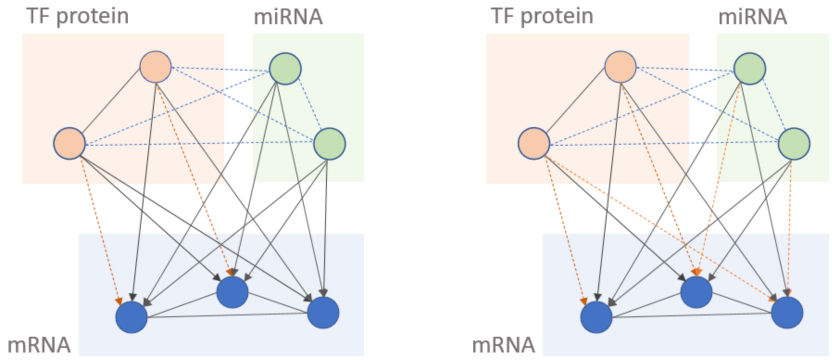

Figure S5. Different levels of regulatory constraints used in the simulation. Left**:** A smaller ratio of masked-out edges (dashed lines). Right**:** A higher ratio of masked-out edges. Blue dashed lines: layer-level constraints that prevent interactions across layers. Orange dashed lines: fine-grained constraints.

Often, we know that certain node pairs should or should not form an edge based on biological knowledge or external experimental evidence. For example, two mRNA molecules that are not transcription factors (TFs) are unlikely to interact directly. Consequently, when constructing the network, we may specify that no edge should form between them. Similarly, if a TF is known to bind a specific list of target mRNAs, edges should not be formed with non-target mRNAs. By

incorporating such constraints, we can improve the accuracy of network inference. One straightforward approach to applying these constraints is post-processing: after estimating a network, we could remove all edges disallowed by the constraints. However, iDDN can integrate constraints directly into the optimization problem, eliminating the need for post-processing. When constraints exclude a substantial proportion of all possible edges, iDDN achieves both higher computational efficiency and greater accuracy.

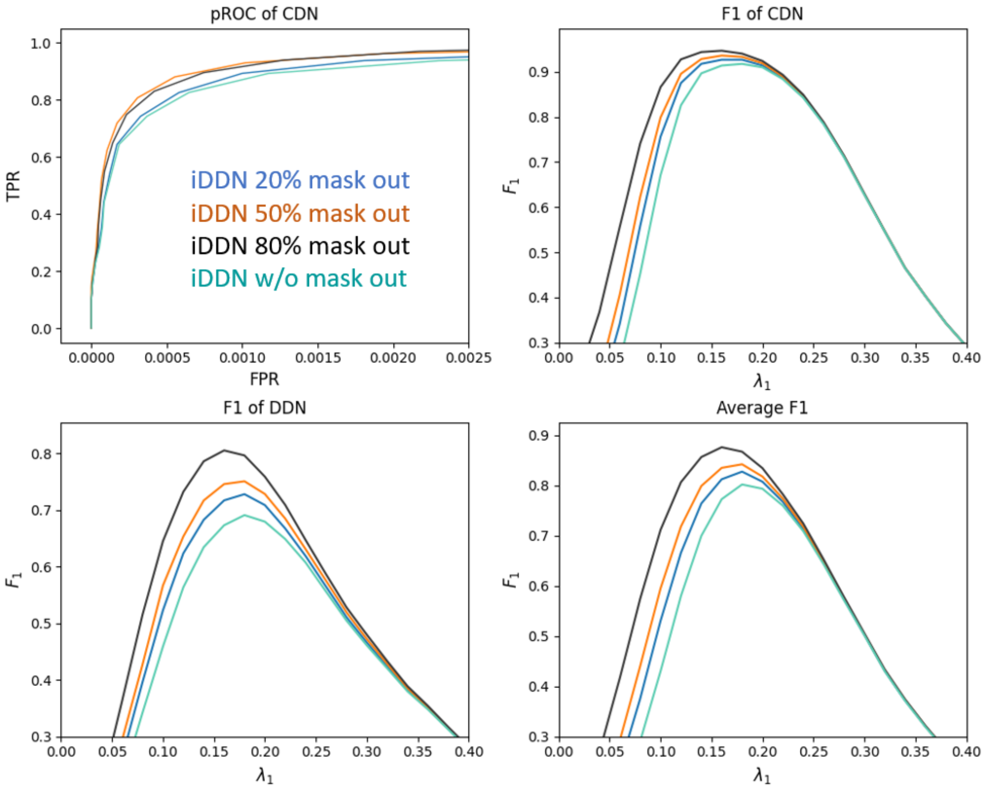

Figure S6. Performance comparison on the effects of incorporating constraints. Each TF or miRNA is connected to two RNAs. The four panels were generated as in Figure S2.

In this simulation, we used the three-layer network introduced in Supplementary Section 2. We assumed that TF proteins and miRNAs do not interact, and no interactions occur among miRNAs (Figure S4, right). Additionally, as shown in Figure S5, certain TF proteins and miRNAs were assumed not to bind specific mRNAs. The impact of different levels of constraints on iDDN's estimation accuracy was investigated by masking out varying ratios of non-true edges. Stronger constraints corresponded to higher masking ratios, and we compared four levels: 0%, 20%, 50%, and 80%. The simulation was performed for two network topologies: one where each regulator connects to two mRNAs (loose connectivity) and another where each regulator connects to five

mRNAs (tight connectivity). Performance evaluation included all within- and between-layer edges, and we assumed the constraints were error-free. In real applications, errors in constraints could reduce accuracy.

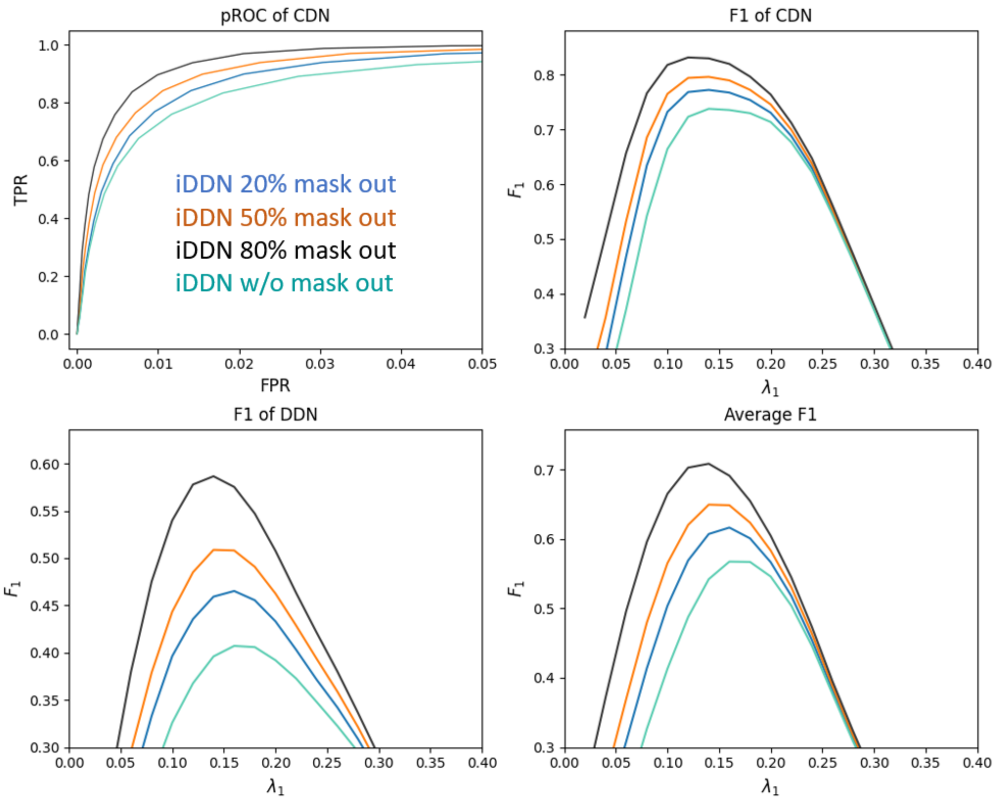

Figure S7. Performance comparison showing the effects of constraints. Here each TF or miRNA is connected to five RNAs. The four panels were obtained the same way as Figure S2.

Figure S6 shows the results when each TF or miRNA connects to two mRNA. The top two panels show that masking out more edges improves the accuracy of the estimated common network. Stronger constraints also enhanced iDDN's performance in estimating differential networks (bottom two panels). Notably, the benefits of using constraints were greater for differential network estimation. When each TF or miRNA connects to five mRNAs (Figure S7), performance improvements due to constraints were larger for both common and differential networks. This is likely because, in more tightly connected layers, constraints help to avoid "confusing" regulatory edges across layers.

3.3 Simulation setup for peer methods

We compared iDDN with two of the most relevant peer methods: JGL (Danaher, et al., 2014) and iDINGO (Class, et al., 2018). JGL is a widely used method for inferring two Gaussian graphical models (GGMs) under two conditions. For our comparison, we used the latest version of the JGL R package available on CRAN (version 2.3.2). Similar to iDDN, JGL requires the specification of two hyperparameters. It is important to note that comparing the two methods using the same set of hyperparameters might be unfair, as the impact of these parameters can differ due to variations in the objective functions of the methods. Therefore, as we did for iDDN, we tested 640 combinations of hyperparameters for JGL. JGL produced two precision matrices, one for each condition. We applied the same thresholding procedure as used with iDDN to convert these matrices into common and differential networks. We also observed that the original JGL code occasionally generated complex values during the eigen-decomposition step, which could result in an error in R and halt execution. To address this issue, we modified the "admm.iter.r" function in the JGL code, ensuring the matrix was treated as symmetric when calling the "eigen" function in R. Further details about the updated JGL code can be found in https://github.com/cbil-vt/iddn_experiments and https://github.com/cbil-vt/jgl_modified.

iDINGO is an extension of DINGO (Ha, et al., 2015) designed to handle multi-omics data. It supports up to three layers in sequential order. In the current simulation, as both the TF protein layer and the miRNA layer regulate the mRNA layer, we combined these two regulatory layers into a single layer in iDINGO and treated the mRNA layer as the second layer. iDINGO builds upon DINGO by first estimating a common network using graphical lasso and then utilizing the remaining signals to estimate differential edges. By default, iDINGO scans a range of λ_1 values when estimating the common network and selects the best network using model selection criteria. In our experiments, we observed that iDINGO frequently failed to choose the optimal λ_1. To ensure a fair comparison, we tested several λ_1 values that performed well in JGL. Since iDINGO estimates differential edges in the form of a score matrix, we applied a range of thresholds to the scores to generate various common and differential networks and evaluated the performance at each threshold. Due to iDINGO's significantly longer runtime compared to iDDN and JGL, we limited the number of bootstrapping steps to 10. Based on our earlier experiments (Fu, et al., 2024), increasing the number of bootstrapping steps did not noticeably affect the performance.

To further ensure fair comparisons with JGL and iDINGO, we incorporated knowledge of constraints into these methods via post-processing. Specifically, all edges prohibited by the constraints were removed. The constrained versions of JGL and iDINGO are referred to as JGL-post and iDINGO-post, respectively. Experiments were repeated 30 times for iDDN and JGL, and 5 times for iDINGO due to its higher computational cost. For each repetition, edges and weights were reassigned, the two conditions were re-generated, and the samples were redrawn.

3.4 Comparative evaluation with peer methods

In Figure S8, we analyzed the performance of iDDN, JGL-post, and iDINGO-post in scenarios where each regulator was connected to two mRNAs. For iDDN and JGL-post, each point represents a specific λ_1 value, with λ_2 optimized to achieve the best F1 score. For iDINGO-post, each point corresponds to a threshold applied to the score matrices. Since these thresholds are not equivalent to λ_1, the iDINGO curves may shift relative to the iDDN and JGL-post curves in the F1 score plots; here, only the peak F1 values are relevant.

The results revealed that iDDN outperformed both JGL-post and iDINGO-post in estimating common and differential networks. Notably, iDINGO performed well in estimating common networks, as it applied graphical lasso to the combined data from both conditions. However, the DINGO algorithm implemented in iDINGO was less effective at detecting differential edges, likely due to its development under different data assumptions (Fu, et al., 2024).

We then analyzed the scenario where each regulator was connected to five mRNAs (Figure S9). A similar trend emerged: iDINGO again performed well in estimating common networks but struggled to detect differential edges. In this case, the performance differences in common network estimation were relatively minor.

Finally, we examined the impact of post-processing steps on the performance of peer methods. iDDN was compared with both JGL without post-processing (denoted as JGL) and JGL with post-processing (JGL-post). Figures S10 and S11 display the results for scenarios where each regulator was connected to two and five mRNAs, respectively. The incorporation of post-processing significantly enhanced the accuracy of JGL in both cases, improving the estimation of both common and differential networks.

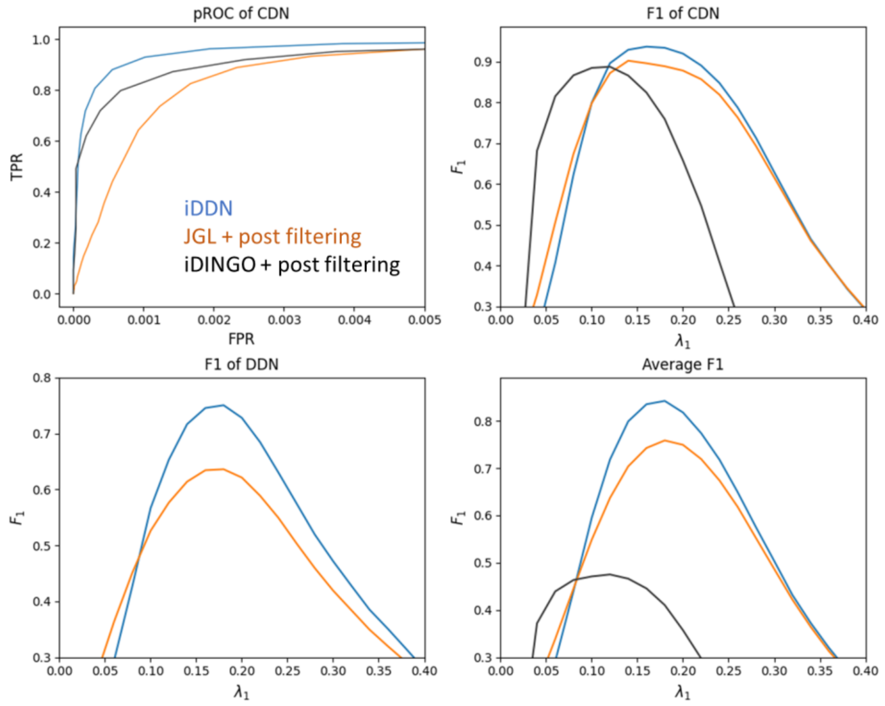

Figure S8. Comparison of iDDN and peer methods with constraints. In this scenario, each TF or miRNA is connected to two RNAs. The four panels were generated in the same manner as in Figure S2.

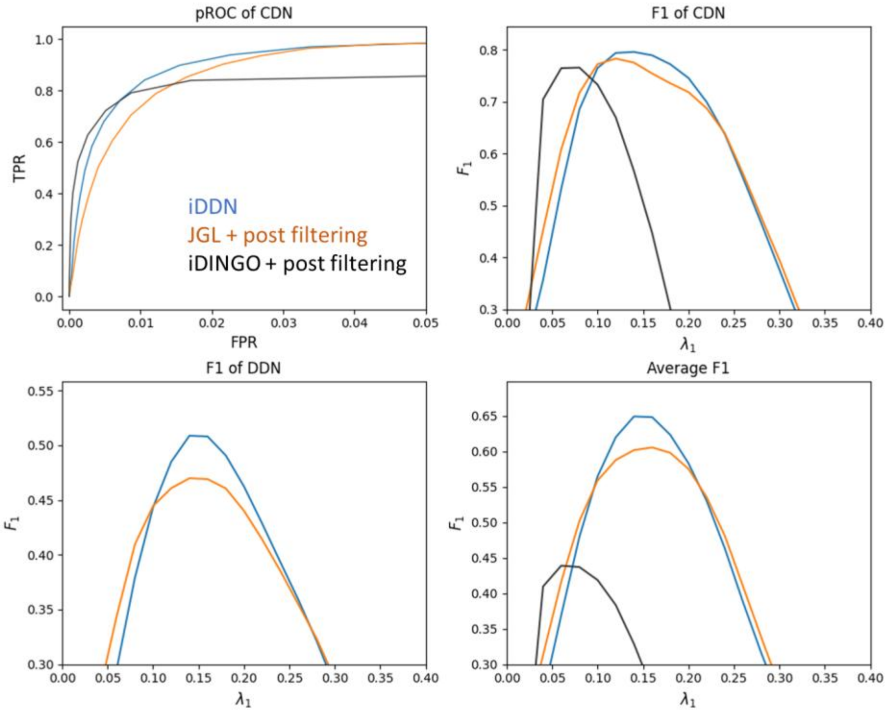

Figure S9. Comparison of iDDN and peer methods with constraints. In this scenario, each TF or miRNA is connected to five RNAs. The four panels were generated in the same manner as in Figure S2.

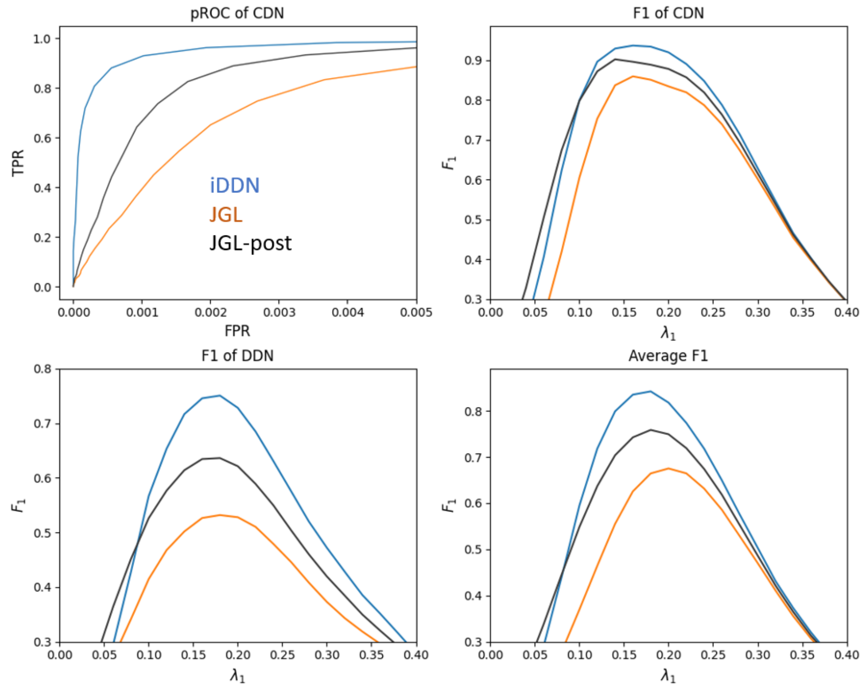

Figure S10. The performance of JGL improves with the post-application of constraints. In this scenario, each TF or miRNA is connected to two RNAs.

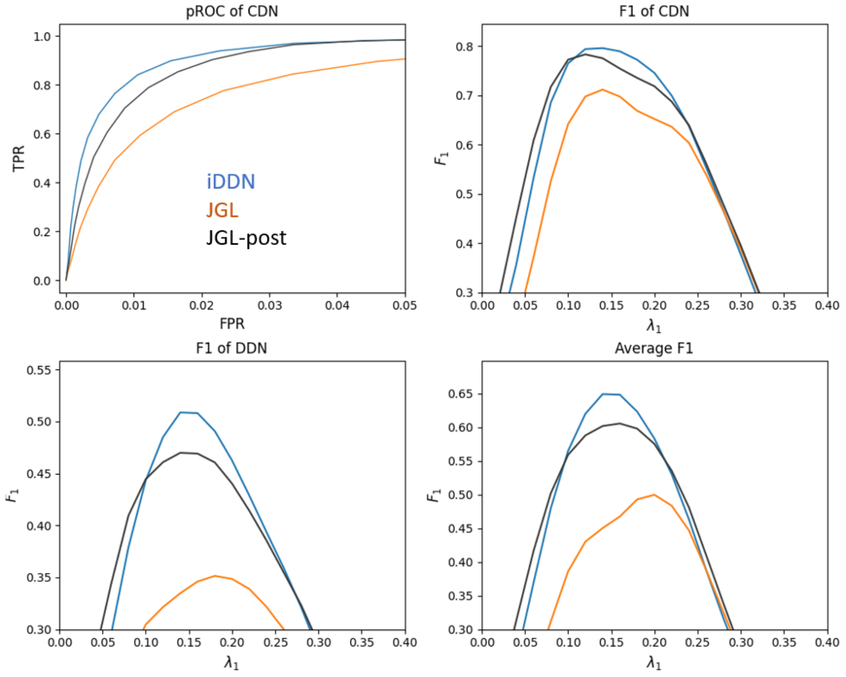

Figure S11. The performance of JGL improves with the post-application of constraints. In this scenario, each TF or miRNA is connected to five RNAs.

4 Comparative evaluation of computational efficiency

To test the runtime and memory usage of iDDN and peer methods, we used a large publicly available single-cell RNA-seq + ATAC-seq dataset from 10x Genomics (10x Genomcis, 2010). The dataset was derived from human peripheral blood mononuclear cells (PBMCs) of a healthy 25-year-old female donor. Details on data acquisition and preprocessing are provided on the data webpage. The raw count data for each omics type was further processed using pipelines from the muon package (Bredikhin, et al., 2022). The resulting RNA-seq data contained 26,349 genes and 10,881 cells across 13 cell types, while the ATAC-seq data comprised 106,086 features and 9,811 cells. We selected two cell types—CD4+ naïve T cells and CD14 monocytes—with sample sizes of 1,513 and 1,241 cells, respectively. Features with a standard deviation below 0.1 in either group were filtered out, leaving 19,654 genes. Of these, 1,180 transcription factors (TFs) were identified based on (Lambert, et al., 2018). To jointly infer TF-target networks for the two cell types, we constrained the iDDN analysis to edges originating only from TF genes.

This section focuses on experiments demonstrating the benefits of using constraints, as well as the impact of sample sizes, feature numbers, and network sparsity. Additional experiments are discussed in Section 3 of the main text. Runtime measurements were obtained using Python's "time" module, while peak memory usage was recorded using the "memory_profiler" package.

We first studied the impact of using constraints by comparing iDDN with DDN. The analysis included all cells in each cell type and the complete set of 19,654 genes. In iDDN, constraints allowed only approximately 5.5% of all possible edges. As shown in Table S2, iDDN completed the task in just 111 seconds, making it 4.5 times faster than DDN. Additionally, iDDN estimated more edges than DDN in the presence of constraints. If similar network sparsity were targeted, iDDN would be even faster. These results demonstrate that incorporating a priori regulator-effector constraints can significantly accelerate the learning process by narrowing the search space to biologically plausible and relevant interactions.

Next, we assessed the runtime and memory usage of iDDN with varying sample sizes. Subsets of cells from each cell type were tested, and to evaluate larger sample sizes, the dataset of 10,881 cells was artificially divided into two groups of 5,440 and 5,441 cells. As shown in Table S3,

iDDN demonstrated efficiency across different sample sizes. It is worth noting that larger sample sizes included more genes in the analysis, as fewer features were filtered out.

We also evaluated the efficiency of iDDN with varying numbers of features, using all cells from each cell type. As illustrated in Table S4, iDDN remained fast and memory-efficient, even when handling a large number of features.

Finally, we examined the impact of the sparsity hyperparameter λ_1 on computational cost. Smaller λ_1 values result in reduced penalties and, consequently, longer runtimes. As shown in Table S5, even with a small λ_1 of 0.01 and high network density (0.45), iDDN maintained low runtime and memory usage, making it suitable for most computing environments.

Table S1. Runtime and memory usage comparison of iDDN, DDN, and JGL on a smaller two-layer network. The analysis was conducted using 378 cells in one group and 310 cells in the other.

**Methods**	**Genes**	**Prior constraints**	**Time (s)**	**Memory (MB)**
iDDN	1000	Yes	7.2	4420
DDN	1000	No	10.76	4491
JGL	1000	No	2685	909
JGL	500	No	386	464
JGL	100	No	5.3	304

Table S2. Running time and memory usage comparison of iDDN and DDN on a larger two-layer network. The analysis was conducted using 1513 cells in one group and 1241 cells in the other. 19654 genes were used.

**Prior constraints**	**Time (s)**	**Memory (MB)**	**Density**	**Edges per group**
Yes (iDDN)	111.1	6976	0.035	814829
No (DDN)	499.1	9280	0.021	477615

Table S3. Impact of sample sizes on iDDN runtime and memory usage. N1 and N2 represent the sample sizes for each group.

**N1**	**N2**	**Time (s)**	**Memory (MB)**	**Density**	**Edges per group**	**Genes**
5440	5441	511.4	11810	0.0069	235716	25658
1513	1241	111.1	6976	0.035	814829	19654
756	620	75.9	6006	0.069	1312001	17233
378	310	58.9	5291	0.090	1363719	15024

Table S4. Impact of gene numbers on iDDN running time and memory usage. 1513 and 1241 cells were used in each group, respectively.

**Genes**	**Time (s)**	**Memory (MB)**	**Density**	**Edges per group**
19654	111.1	6976	0.035	814829
9827	29.9	5681	0.040	231115
4913	12.7	4920	0.044	61004

Table S5. Impact of network sparsity on iDDN running time and memory usage. 1513 and 1241 cells were used in each group, along with 19654 genes.

$\boldsymbol{\lambda_1}$	**Time (s)**	**Memory (MB)**	**Density**	**Edges per group**
0.1	77.3	7001	0.004	99508
0.05	111.1	6976	0.035	814829
0.03	155.5	6982	0.113	2610429
0.01	274.8	7091	0.454	10529470

Table S6. Runtime and memory usage of iDDN on a three-layer network with two omics types. The analysis includes 1,344 and 1,096 cells in each group, with 19,131 mRNAs and 90,451 ATAC sites. This table serves as an extension of Table 1 in the main text.

$\boldsymbol{\lambda_1}$	**Time (s)**	**Memory (GB)**	**Density**	**Edges in T cell**	**Edges in monocyte**
0.025	1718	20.6	0.1185	20551986	25555240
0.05	1669	19.4	0.0299	5092228	6577825
0.1	1626	19.1	0.0046	870513	910200

5 Ablation studies of running time and inference accuracy

We designed two ablation studies to evaluate the impact of iDDN's new functions on runtime and inference accuracy. The studies utilized the same three-layer synthetic network introduced in Supplementary Section 2.1, with 200 samples generated for each condition. In this analysis, we used λ_1=0.15 and λ_2=0.05, which had demonstrated strong performance in prior simulation studies. Each experiment was repeated 50 times.

The first study assessed the impact of iDDN's key functions on runtime (Table S7). We used DDN 3.0 with the residual update strategy as the baseline. Parallel computing functions were implemented using the "Joblib" Python package. "Layer-level constraints" introduced relationships between specific layers; for instance, the TF protein layer was allowed to regulate the RNA layer. "Detailed prior" incorporated prior knowledge to specify permissible edges between individual molecules. In this experiment, 80% of edges known to be false were masked out (Supplementary Section 3). As shown in Table S7, the use of parallel computing and the integration of layer-level and prior constraints significantly improved computational efficiency. Memory usage was not measured, as the network in this study was small. Four CPU cores were utilized.

The second study aimed to demonstrate how iDDN's new functions improve network inference accuracy (see Table S8 for the common network and Table S9 for the differential network). Baseline results were obtained by inferring two separate Gaussian graphical models using Lasso. Then a similarity penalty, as implemented in DDN 3.0, was applied to encourage similarity between networks under the two conditions. For each case, we used $\lambda_1 = 0.15$, and $\lambda_2 = 0.05$, and calculated the precision, recall, and F1 scores for both the common and differential networks, averaged over 50 repetitions. Our results demonstrated that the F1 score improved with the inclusion of iDDN's new functions. However, since only one set of hyperparameters was tested, precision and recall did not consistently improve across all cases. We emphasize that the F1 score provides a more comprehensive measure of performance. In practical applications, multiple combinations of these hyperparameters should be explored, and the optimal network is typically selected by users based on domain-specific knowledge.

Table S7. Impact of new functions in iDDN on running time

Ablation steps	Running time
Baseline (DDN3.0)	27.9
Baseline + parallel	14.1
Baseline + parallel + layer level constraints	5.30
Baseline + parallel + layer level constraints + detailed prior	1.91

Table S8. Impact of new functions in iDDN on common network inference.

Ablation steps	Precision	Recall	F1
Baseline (Separate learning by Lasso)	0.895	0.501	0.641
Baseline + similarity penalty (DDN3.0)	0.688	0.775	0.727
Baseline + similarity penalty + layer level constraints	0.730	0.778	0.751
Baseline + similarity penalty + layer level constraints + detailed prior	0.831	0.787	0.807

Table S9. Impact of new functions in iDDN on differential network inference.

Ablation steps	Precision	Recall	F1
Baseline (Separate learning by Lasso)	0.172	0.680	0.274
Baseline + similarity penalty (DDN3.0)	0.398	0.397	0.397
Baseline + similarity penalty + layer level constraints	0.473	0.400	0.432
Baseline + similarity penalty + layer level constraints + detailed prior	0.650	0.408	0.501

6 Analysis of GPAA human arterial data

Here we provide additional details regarding the data pre-processing and feature selection for GPAA data analysis. Other aspects of this analysis are presented in Section 4.2 of the main text. Ideally, the networks should be constructed using the protein form of transcription factors (TFs) and the mRNA form of signature genes (SGs). However, due to the low expression levels of TFs in the proteomics data, they could not be reliably utilized. Therefore, we used the mRNA expression of TFs as a proxy to construct a two-layer network between TFs and SG mRNAs.

For latent feature 2 (LF2), we used a list of 100 SGs from LISA (Qin, et al., 2020) to identify TFs that might regulate these SGs. Of the top TFs returned by LISA, 130 were also present in the GPAA mRNA dataset. The samples were divided into two groups: NL (normal, 29 samples) and FP (disease, 37 samples). Edges were restricted to those among TFs and between TFs and mRNAs.

Bootstrapping was performed 100 times, and iDDN was applied to each bootstrapped sample. Based on these 100 iterations, the coefficient of variance (CV) was calculated and used to filter out edges with high uncertainty (threshold = 0.8). Hyperparameters λ_1 and λ_2 were set to 0.1 and 0.04, respectively, to enhance the interpretability of the results by avoiding overly dense networks while yielding a reasonable number of hubs.

Table S10. Hub TFs in the GPAA common network. Only TFs with degree ≥ 6 are shown.

TF	Degree
SOX10	20
CEBPA	13
SOX2	9
SREBF2	8
TEAD1	8
KLF12	8
KLF5	7
TFAP2A	7
HIF1A	7
KLF9	6
SMAD3	6
KLF15	6
RUNX2	6
PBX3	6
FOSL1	6
EBF3	6
MAX	6

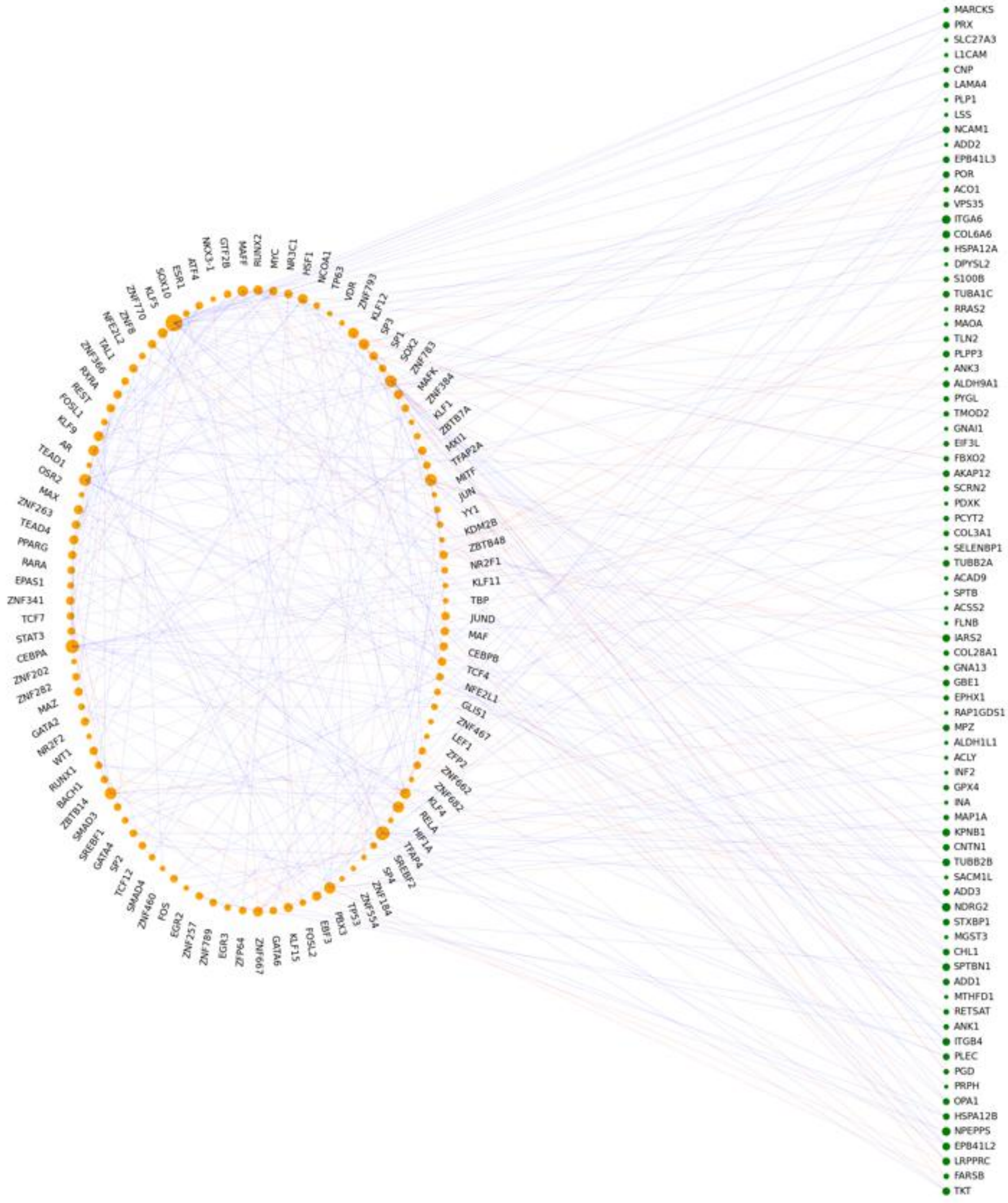

Figure S12. The common network inferred by iDDN for the GPAA data. Transcription factor (TF) nodes are displayed on the left (in orange), while signature gene (SG) mRNA nodes are shown on the right (in green). The size of each node reflects its degree. Blue edges represent positive dependency values, whereas red edges indicate negative dependencies.

7 Analysis of phosphorylation data from CPTAC

We applied iDDN to the CPTAC phosphorylation data, which includes LC-MS/MS-quantitated expression of kinases and HuProt array-based activity quantitation of substrates (Song, et al., 2019). The dataset comprises 108 ovarian tumor samples, a subset of the TCGA high-grade serous ovarian carcinoma (HGSC) specimens, collected from newly diagnosed HGSC patients. For these samples, PTM signatures were profiled on HuProt arrays, and tyrosine (Tyr) phosphorylation signals were tested using ovarian tumor lysates on the same arrays. For the observed Tyr phosphorylation, 54 kinases and 118 corresponding substrates were identified, forming 245 potential kinase-substrate interactions (KSRs) in ovarian cancers (Hu, et al., 2014).

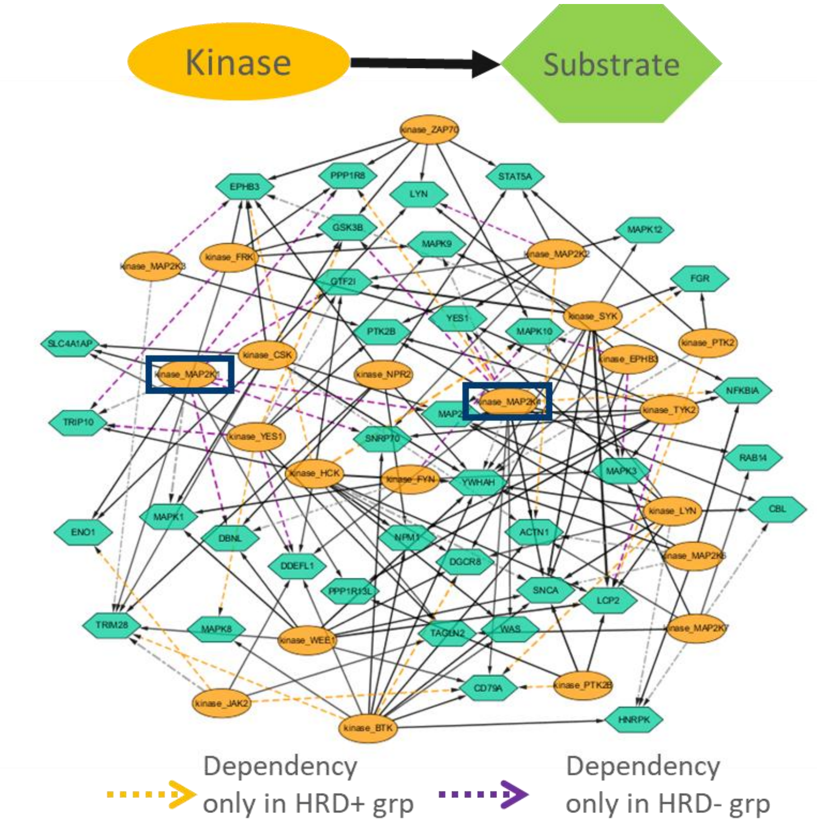

Figure S13. The dependency network between protein kinases and substrates detected by iDDN. Black solid lines represent edges common to both the HRD+ and HRD- groups, while yellow and purple dashed lines indicate edges specific to each condition. In this model, the focus is solely on edges from kinases to substrates, reflecting a biologically meaningful constraint.

In this study, protein kinases were treated as regulators influencing the expression of substrate molecules. To further refine the analysis, we constrained inter-omics rewiring to interactions between regulators and their targets. Additionally, edges were restricted to those listed in the kinase-substrate relationship (KSR) database from PhosphoNetwork (Hu et al., 2014). Using these constraints, iDDN was applied to reconstruct phosphorylation networks.

Figure S13 illustrates the iDDN results for both common and differential networks. Multiple rewiring events in KSRs were identified between the HRD+ and HRD- groups. Notably, two kinases, PTK2 and PTK2B, were associated with significant rewiring events and have previously been shown to be dysregulated in ovarian cancer cell line models (Song et al., 2019).

8 Analysis of single-cell PBMC data

In this section, we discuss the accuracy of iDDN in inferring cell-type-specific gene regulatory networks (GRNs) using the same publicly available single-cell ATAC+RNA sequencing dataset (10x Genomcis, 2010) introduced in Supplementary Section 4. Additionally, we present a simple case study on the differential hub nodes between T cells and monocytes.

8.1 Evaluation of iDDN cell-type specific GRN inference

Our evaluation steps followed the methodology outlined in LINGER (Yuan and Duren, 2024). However, in this study, we only utilized scRNA-seq data and did not integrate ATAC-seq data. As demonstrated by (Yuan and Duren, 2024), successful integration of scRNA-seq and ATAC-seq data requires careful consideration of the distinct properties of each data type, along with sophisticated modeling strategies. A direct and naïve application of iDDN is unlikely to achieve competitive results compared to tools highly optimized for this specific data integration task.

We selected 1,513 CD4+ naïve T cells and 1,241 CD14 monocytes from the PBMC scRNA-seq dataset. After filtering out mRNAs with variance lower than 0.1 in either condition, 19,654 mRNAs remained. Among these, based on Lambert et al. (2018), we identified 1,180 transcription factors (TFs). ChIP-seq data for these two cell types in humans served as the ground truth. Following (Yuan and Duren, 2024), we used the Cistrome database (Zheng, et al., 2019), which included five ChIP-seq datasets for monocytes ("STAT1," "IRF1," "CTCF," "RUNX1," and "SPI1") and four for T cells ("ETS1," "RUNX1," "FOXP3," and "REST"). Consequently, our evaluation of iDDN performance focused on these nine TFs. For each ChIP-seq dataset, target scores were calculated using BETA (Wang, et al., 2013) from the Cistrome processing pipeline. A threshold of 1.5 was applied to prioritize clearer binding signals while avoiding an excessive number of targets. Using this threshold, the five TFs in monocytes had more targets than the four TFs in T cells. This trend was also observed for RUNX1, the only TF with ChIP-seq data available for both cell types.

We used iDDN to jointly infer networks between TF mRNAs and non-TF mRNAs for the two cell types. Each TF was allowed to regulate any molecule except itself, while non-TF mRNAs were not permitted to regulate any molecule. Results were obtained using three different values of λ_1, with λ_2 fixed at 0.001. For each λ_1 value, we recorded precision, recall, and F1 scores for each

TF. These results are presented in Tables S11, S12, and S13. In these tables, the top five rows correspond to TFs for monocytes, while the bottom four rows correspond to TFs for T cells, with shading used to distinguish the groups.

From these results, we observe that without incorporating ATAC-seq information, achieving strong consistency with ChIP-seq data is challenging. These findings align with the consensus in the field, which suggests that GRN inference using only scRNA-seq data often performs no better than random guessing, as highlighted in various benchmark studies (Marbach, et al., 2012; Pratapa, et al., 2020), as well as in (Yuan and Duren, 2024). Even when scATAC-seq data is integrated, significant improvements are not consistently observed (Yuan and Duren, 2024). Therefore, we conclude that comparing iDDN with existing methods requires (1) substantial extensions to iDDN to better account for the unique properties of data types involved in GRN inference and (2) the availability of higher-quality, cell-type-specific ground-truth data for a broader set of TFs. Consequently, peer method comparisons were not conducted here.

Table S11. iDDN accuracy for each TF. λ_1=0.05, λ_2=0.001.

**TF**	**Number**	**Edges**	**Precision**	**Recall**	**F1**
STAT1	6786	1082	0.430	0.068	0.118
IRF1	4900	942	0.369	0.071	0.119
CTCF	3816	800	0.223	0.046	0.077
RUNX1	8595	1018	0.520	0.061	0.110
SPI1	113	1189	0.013	0.141	0.024
ETS1	2528	748	0.207	0.061	0.094
RUNX1	617	660	0.042	0.045	0.043
FOXP3	372	658	0.013	0.024	0.017
REST	701	602	0.036	0.031	0.033

Table S12. iDDN accuracy for each TF. λ_1=0.03, λ_2=0.001.

**TF**	**Number**	**Edges**	**Precision**	**Recall**	**F1**
STAT1	6786	3715	0.377	0.206	0.267
IRF1	4900	3596	0.279	0.205	0.236
CTCF	3816	3383	0.200	0.177	0.188
RUNX1	8595	3704	0.461	0.198	0.277
SPI1	113	3766	0.008	0.274	0.015
ETS1	2528	3262	0.147	0.190	0.166
RUNX1	617	3073	0.038	0.191	0.063
FOXP3	372	1725	0.023	0.110	0.039
REST	701	2985	0.039	0.166	0.063

Table S13. iDDN accuracy for each TF. λ_1=0.01, λ_2=0.001.

TF	Number	Edges	Precision	Recall	F1
STAT1	6786	10715	0.354	0.560	0.434
IRF1	4900	10665	0.252	0.550	0.346
CTCF	3816	10706	0.197	0.553	0.290
RUNX1	8595	10815	0.442	0.556	0.493
SPI1	113	10569	0.006	0.592	0.012
ETS1	2528	10651	0.132	0.556	0.213
RUNX1	617	10702	0.031	0.538	0.058
FOXP3	372	8089	0.022	0.486	0.042
REST	701	10661	0.035	0.539	0.066

8.2 Case study: three-layer TF-ATAC-mRNA network

When the expression of a TF is highly correlated with the chromatin accessibility score of a specific ATAC site, we hypothesize that this TF is more likely to have a causal regulatory relationship with the genes associated with that ATAC site (Cao, et al., 2018). Conversely, if a TF is only highly correlated with the expression of a gene but the chromatin accessibility remains low, the relationship between the TF and the gene may not be direct. Instead, the high correlation could be attributed to indirect effects mediated through other genes.

Based on this reasoning, we created a three-layer network using scRNA-seq and scATAC-seq data obtained from the same cells (Figure S14A). The combined RNA and ATAC data included 1,344 T cells and 1,098 monocytes, along with 19,131 mRNAs (of which 1,163 were TFs) and 90,451 ATAC sites. The first layer of the network represents TF mRNAs, the second represents ATAC sites, and the third includes all mRNAs. Each TF was allowed to connect to any ATAC site, reflecting their ability to move within cellular space. For each ATAC site, we restricted connections to mRNAs on the same chromosome, as their effects are typically localized. To enhance precision, it is often preferable to limit the distance between ATAC sites and related genes, such as within 1 million base pairs. This network design extends the analysis of (Cao, et al., 2018) by incorporating additional layers and jointly analyzing two cell types.

We applied iDDN with λ_1 = 0.1 and λ_2 = 0.005, focusing on the rewiring of edges from TFs to ATAC sites. The number of differential edges for each TF was calculated (Figure S14B), and the top TFs with the largest number of differential edges are shown in Figure S14C. For example, CEBPE had 950 edges in T cells and 1,810 edges in monocytes, resulting in 2,712 differential

edges. This indicates that CEBPE exhibits markedly different wiring patterns to ATAC sites between the two cell types. CEBPE is known to be highly expressed in monocytes, where it plays a critical role in their proper development, but it is not a major regulator of T cell functions (Huber, et al., 2012; Laiosa, et al., 2006; Tamura, et al., 2017).

In future work, similar analyses can be conducted to examine the relationships between ATAC sites and their cis-regulatory targets, as well as to explore the complete regulatory path from TFs to ATAC sites to target genes based on the inferred networks.

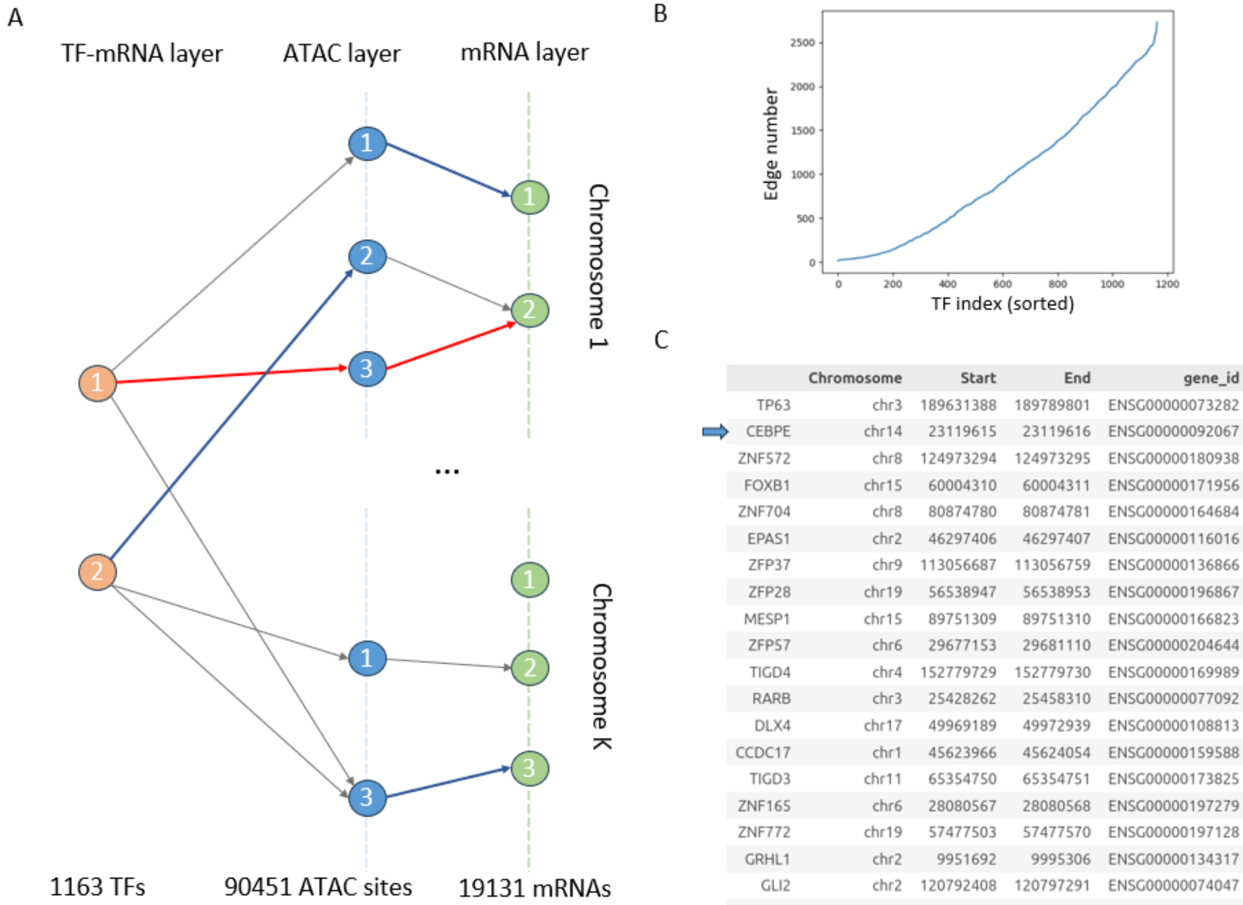

	Chromosome	Start	End	gene_id
TP63	chr3	189631388	189789801	ENSG00000073282
CEBPE	chr14	23119615	23119616	ENSG00000092067
ZNF572	chr8	124973294	124973295	ENSG00000180938
FOXB1	chr15	60004310	60004311	ENSG00000171956
ZNF704	chr8	80874780	80874781	ENSG00000164684
EPAS1	chr2	46297406	46297407	ENSG00000116016
ZFP37	chr9	113056687	113056759	ENSG00000136866
ZFP28	chr19	56538947	56538953	ENSG00000196867
MESP1	chr15	89751309	89751310	ENSG00000166823
ZFP57	chr6	29677153	29681110	ENSG00000204644
TIGD4	chr4	152779729	152779730	ENSG00000169989
RARB	chr3	25428262	25458310	ENSG00000077092
DLX4	chr17	49969189	49972939	ENSG00000108813
CCDC17	chr1	45623966	45624054	ENSG00000159588
TIGD3	chr11	65354750	65354751	ENSG00000173825
ZNF165	chr6	28080567	28080568	ENSG00000197279
ZNF772	chr19	57477503	57477570	ENSG00000197128
GRHL1	chr2	9951692	9995306	ENSG00000134317
GLI2	chr2	120792408	120797291	ENSG00000074047
CTCFL	chr20	57524256	57525652	ENSG00000124092

Figure S14. Application of iDDN on PBMC data. (A) A cartoon illustration of the three-layer network design. Red edges represent those that exist only in T cells, blue edges correspond to those found exclusively in monocytes, and grey edges are shared between both conditions. (B) The number of differential edges for each TF, sorted in ascending order. (C) The top differential hub TFs and their corresponding chromosomal locations.

9 Additional discussions

9.1 Novelties of iDDN and relationship with peer methods.

Various methods have been developed to infer the network structures from biological data. Among them, DDN3.0 (Fu, et al., 2024), JGL (Danaher, et al., 2014), and iDINGO (Class, et al., 2018) are closely related to our iDDN work. However, these methods generally lack the flexibility to effectively integrate data from multiple omics types. While it is feasible to concatenate data from different types, this approach often overlooks biological knowledge about the networks (e.g., the central dogma), which may lead to suboptimal results.

Specifically, compared to JGL, iDDN incorporates layer structures and biologically plausible prior constraints to improve accuracy and efficiency. JGL does not account for layers and can only apply constraints post hoc, which reduces accuracy and introduces unnecessary computational overhead. Additionally, JGL is significantly slower, particularly for datasets with more than 1,000 features, and it does not support parallel computation. Compared with DDN3.0, iDDN enables the specification of layers and constraints, facilitating the modeling of multi-omics data. These novel capabilities of iDDN enhance accuracy, efficiency, and flexibility, as demonstrated in our simulation studies and real-world applications.

Very few methods can utilize data from multiple omics types to estimate differential networks. One such example is iDINGO, which was developed based on DINGO to integrate miRNA, RNA, and proteins under Markov assumptions. However, iDINGO models only the unidirectional flow from miRNA → RNA → protein. While this is useful in certain scenarios, iDINGO lacks the flexibility required to address tasks involving the integration of diverse omics data types and the incorporation of prior constraints. For instance, explicitly modeling the regulation of TF proteins on mRNA is not feasible with iDINGO. Furthermore, iDINGO was not designed to jointly estimate common and differential networks, which can result in suboptimal outcomes. Its computational cost is also significantly higher, further limiting its practicality for large-scale or complex analyses.

For completeness, when comparing iDDN with GRN inference methods like GINEI3 (Huynh-Thu, et al., 2010), SCENIC+ (Bravo Gonzalez-Blas, et al., 2023), and LINGER (Yuan and Duren, 2024), iDDN stands out by enabling joint inference of two conditions, thereby improving the accuracy of common and differential network inference. Furthermore, these GRN methods are

typically task-specific, lacking the general applicability and flexibility of iDDN. Compared with popular co-expression network inference methods like WGCNA (Langfelder and Horvath, 2008), iDDN is based on Gaussian graphical models (GGM). As a result, iDDN can better distinguish direct and indirect edges, often resulting in improved accuracy.

9.2 Our preference for moderately sized multi-omics datasets

Our real-world applications primarily focus on networks with moderate sample sizes and feature counts. Regarding the objective of iDDN, we clarify that, in real-data applications, iDDN modeling identifies a focused and complex molecular regulatory circuitry as a network of differentially connected molecular entities. Such networks are typically used to assist in the inference of potential key pathways and regulators. These networks provide valuable frameworks for constructing and verifying mechanistic models, which help to offer plausible data interpretations, gain new insights into disease biology, and generate hypotheses for further experimental validation and investigation (Herrington, et al., 2018; Zhang, et al., 2016).

For example, while the statistical associations predicted by our model cannot distinguish between drivers and consequences of HRD status, the observed enrichment of proteins associated with histone acetylation motivated us to identify and quantify acetylated peptides from our CPTAC global proteomic data. Comparative analysis of 399 acetylated peptides identified 15 with significant differences between HRD and non-HRD samples, including dual acetylation at K12 and K16 of histone H4. Acetylation of H4 is involved in the choice of DNA double-strand break (DSB) repair pathways (homologous recombination or non-homologous end joining), a process partially regulated by HDAC1.

These findings were verified using independent samples and technical approaches. The combined observations of increased HDAC1 levels (a hub protein also identified by our model) and associated pathway-level proteins, along with decreased acetylation of H4 in HRD patients at the post-translational modification (PTM) level, offer insights into the potential role of HDAC1 in modulating the choice of DSB repair pathways (Zhang, et al., 2016).

Although many biological problems of interest involve datasets with limited size, we have benchmarked the runtime and memory usage of iDDN across datasets of varying sizes to demonstrate its applicability and efficiency. These benchmarks also illustrate how performance scales with the number of omics layers and network sparsity.

9.3 Realities in modeling non-linear effects

We clarify that while iDDN employs linear models for each condition, the combination of common and rewired networks inferred by iDDN enables it to capture a significant form of nonlinear regulatory interactions across different biological conditions. This capability is supported by two recent studies that focus on the nonlinearity of regulation in biological networks. Specifically, iDDN identifies the canalization effect of regulatory relationships in biological networks, such as the "transistor switch-type" nonlinearity (Kadelka and Murrugarra, 2024; Manicka, et al., 2023).

The study by Manicka et al. comprehensively analyzed 137 published Boolean network models, which included a variety of complex nonlinear regulatory interactions. Their comparisons of these biological models revealed that biological regulation tends to be less nonlinear than anticipated. This observation suggests a possible evolutionary selection pressure for biological systems to favor linear regulation on average, while diseases, conversely, may selectively evolve nonlinear rules (Manicka et al., 2023). A follow-up study by Kadelka et al. further demonstrated that biological networks are enriched for certain common network structures and are more canalizing than expected, indicating that biological networks are better approximated by linear and canalizing models (Kadelka and Murrugarra, 2024).

We acknowledge that nonlinear methods may perform better for certain networks (Marbach, et al., 2012). However, iDDN was designed to complement these methods, rather than replace them. Gene regulatory network benchmarks have shown that nonlinear models do not consistently outperform linear models across various network types and omics data. This is partly because true nonlinear effects are difficult to capture without risking overfitting. Moreover, these methods were all developed for single-condition analyses and are unable to jointly infer networks under two conditions, which is the primary focus of iDDN.

9.4 Considerations with data with high noise and/or missing rate

Handling datasets with high missing rates and high noise levels can be challenging. Concerning high-noise datasets, in addition to quality control and preprocessing (Herrington, et al., 2018; Zhang, et al., 2016), iDDN uses biologically plausible constraints to reduce spurious dependencies by incorporating a priori knowledge or additional data (e.g., TF-binding, ATAC-seq) when

available. Concerning datasets with high missing rates, we suggest a three-staged approach to address this issue: 1) eliminate features or samples with significant missing rates (Herrington, et al., 2018), 2) perform a mechanism-integrated group-wise pre-imputation to retain informative missingness when group information is available (Du, et al., 2024), and 3) implement an overall imputation step following the best practices (Shen, et al., 2022).

We note that iDDN package itself does not contain functions for handling missing values.

9.5 Considerations on working on specific omics types and tasks.

We emphasize that iDDN was developed as a generally applicable, flexible, and easy-to-use tool for modeling multi-omics biological networks under two conditions. For specific data types and particular biological problems, we recommend that readers explore specialized tools and evaluate their performance using appropriate ground truth data. For instance, in the case of GRN inference on scRNA-seq + scATAC-seq data, LINGER (Yuan and Duren, 2024) may be a better choice than directly applying iDDN. This is primarily because iDDN is not specifically designed or optimized for these data types, nor does it account for their unique properties.

Nevertheless, iDDN offers a straightforward and effective option for initial data analysis, especially when tools designed for specific data types are less user-friendly. For example, LINGER requires retraining its neural network on the user's data, which can present challenges. In contrast, the flexibility and efficiency of iDDN allow users to experiment with various approaches to multi-omics network analysis. As demonstrated in our application to PBMC data, iDDN enables users to investigate the relationship between TFs and ATAC sites under appropriate location constraints, revealing significant rewiring between two cell types.

Moreover, our applications of iDDN on real bulk data highlight its capability to identify TFs that play crucial roles in disease progression. This feature also provides some justification for our choice to incorporate "transistor switch-type" nonlinearity.

References

10x Genomcis. PBMC from a healthy donor - granulocytes removed through cell sorting (10k). In.; 2010.

Barabasi, A.L. and Albert, R. Emergence of scaling in random networks. Science 1999;286(5439):509-512.

Bravo Gonzalez-Blas, C., et al. SCENIC+: single-cell multiomic inference of enhancers and gene regulatory networks. Nat Methods 2023;20(9):1355-1367.

Bredikhin, D., Kats, I. and Stegle, O. MUON: multimodal omics analysis framework. Genome Biol 2022;23(1):42.

Cao, J., et al. Joint profiling of chromatin accessibility and gene expression in thousands of single cells. Science 2018;361(6409):1380-1385.

Class, C.A., et al. iDINGO-integrative differential network analysis in genomics with Shiny application. Bioinformatics 2018;34(7):1243-1245.

Danaher, P., Wang, P. and Witten, D.M. The joint graphical lasso for inverse covariance estimation across multiple classes. J R Stat Soc Series B Stat Methodol 2014;76(2):373-397.

Du, D., et al. Embracing the informative missingness and silent gene in analyzing biologically diverse samples. Sci Rep 2024;14(1):28265.

Fu, Y., et al. DDN3.0: Determining significant rewiring of biological network structure with differential dependency networks. Bioinformatics 2024.

Ha, M.J., Baladandayuthapani, V. and Do, K.A. DINGO: differential network analysis in genomics. Bioinformatics 2015;31(21):3413-3420.

Harris, C.R., et al. Array programming with NumPy. Nature 2020;585(7825):357-362.

Herrington, D.M., et al. Proteomic Architecture of Human Coronary and Aortic Atherosclerosis. Circulation 2018;137(25):2741-2756.

Hu, J., et al. PhosphoNetworks: a database for human phosphorylation networks. Bioinformatics 2014;30(1):141-142.

Hu, J.X., Thomas, C.E. and Brunak, S. Network biology concepts in complex disease comorbidities. Nat Rev Genet 2016;17(10):615-629.

Huber, R., et al. Regulation of C/EBPbeta and resulting functions in cells of the monocytic lineage. Cell Signal 2012;24(6):1287-1296.

Huynh-Thu, V.A., et al. Inferring regulatory networks from expression data using tree-based methods. PLoS One 2010;5(9).

Kadelka, C. and Murrugarra, D. Canalization reduces the nonlinearity of regulation in biological networks. NPJ Syst Biol Appl 2024;10(1):67.

Laiosa, C.V., et al. Reprogramming of committed T cell progenitors to macrophages and dendritic cells by C/EBP alpha and PU.1 transcription factors. Immunity 2006;25(5):731-744.

Lam, S.K., Pitrou, A. and Seiber, S. Numba: A llvm-based python jit compiler. In, Proceedings of the Second Workshop on the LLVM Compiler Infrastructure in HPC. 2015.

Lambert, S.A., et al. The Human Transcription Factors. Cell 2018;172(4):650-665.

Langfelder, P. and Horvath, S. WGCNA: an R package for weighted correlation network analysis. BMC Bioinformatics 2008;9:559.

Manicka, S., et al. The nonlinearity of regulation in biological networks. NPJ Syst Biol Appl 2023;9(1):10.

Marbach, D., et al. Wisdom of crowds for robust gene network inference. Nat Methods 2012;9(8):796-804.

Mitra, K., et al. Integrative approaches for finding modular structure in biological networks. Nat Rev Genet 2013;14(10):719-732.

Pratapa, A., et al. Benchmarking algorithms for gene regulatory network inference from single-cell transcriptomic data. Nat Methods 2020;17(2):147-154.

Qin, Q., et al. Lisa: inferring transcriptional regulators through integrative modeling of public chromatin accessibility and ChIP-seq data. Genome Biol 2020;21(1):32.

Shen, M., et al. Comparative assessment and novel strategy on methods for imputing proteomics data. Sci Rep 2022;12(1):1067.

Song, G., et al. Proteome-wide Tyrosine Phosphorylation Analysis Reveals Dysregulated Signaling Pathways in Ovarian Tumors. Mol Cell Proteomics 2019;18(3):448-460.

Tamura, A., et al. C/EBPbeta is required for survival of Ly6C(-) monocytes. Blood 2017;130(16):1809-1818.

Tian, Y., et al. Knowledge-fused differential dependency network models for detecting significant rewiring in biological networks. BMC Syst Biol 2014;8(1):87.

Wang, S., et al. Target analysis by integration of transcriptome and ChIP-seq data with BETA. Nat Protoc 2013;8(12):2502-2515.

Yuan, Q. and Duren, Z. Inferring gene regulatory networks from single-cell multiome data using atlas-scale external data. Nat Biotechnol 2024.

Zhang, B., et al. Differential Dependency Network Analysis to Identify Condition-Specific Topological Changes in Biological Networks. Bioinformatics 2009;25(4):526-532.

Zhang, B. and Wang, Y. Learning structural changes of Gaussian graphical models in controlled experiments. In, Uncertainty in Artificial Intelligence (UAI 2010). 2010.

Zhang, H., et al. Integrated Proteogenomic Characterization of Human High-Grade Serous Ovarian Cancer. Cell 2016;166(3):755-765.

Zhao, T., et al. The huge Package for High-dimensional Undirected Graph Estimation in R. J Mach Learn Res 2012;13:1059-1062.

Zheng, R., et al. Cistrome Data Browser: expanded datasets and new tools for gene regulatory analysis. Nucleic Acids Res 2019;47(D1):D729-D735.
